# Targeting the m^6^A RNA methyltransferase METTL3 attenuates the development of kidney fibrosis

**DOI:** 10.1038/s12276-024-01159-5

**Published:** 2024-02-01

**Authors:** Hae Rim Jung, Jeonghwan Lee, Seung-Pyo Hong, Nayeon Shin, Ara Cho, Dong-Jin Shin, Jin Woo Choi, Jong-Il Kim, Jung Pyo Lee, Sung-Yup Cho

**Affiliations:** 1 Genomic Medicine Institute, Medical Research Center, Seoul National University, Seoul, Republic of Korea; 2 Department of Internal Medicine, Seoul National University College of Medicine, Seoul, Republic of Korea; 3 Department of Internal Medicine, Seoul National University Boramae Medical Center, Seoul, Republic of Korea; 4 Department of Biomedical Sciences, Seoul National University College of Medicine, Seoul, Republic of Korea; 5 Medicine Major, Seoul National University College of Medicine, Seoul, Republic of Korea; 6 College of Pharmacy, Kyung Hee University, Seoul, Republic of Korea; 7 Cancer Research Institute, Seoul National University, Seoul, Republic of Korea

**Keywords:** RNA modification, Chronic kidney disease

## Abstract

Kidney fibrosis is a major mechanism underlying chronic kidney disease (CKD). N^6^-methyladenosine (m^6^A) RNA methylation is associated with organ fibrosis. We investigated m^6^A profile alterations and the inhibitory effect of RNA methylation in kidney fibrosis in vitro (TGF-β-treated HK-2 cells) and in vivo (unilateral ureteral obstruction [UUO] mouse model). METTL3-mediated signaling was inhibited using siRNA in vitro or the METTL3-specific inhibitor STM2457 in vivo and in vitro. In HK-2 cells, METTL3 protein levels increased in a dose- and time-dependent manner along with an increase in the cellular m^6^A levels. In the UUO model, METTL3 expression and m^6^A levels were significantly increased. Transcriptomic and m^6^A profiling demonstrated that epithelial-to-mesenchymal transition- and inflammation-related pathways were significantly associated with RNA m^6^A methylation. Genetic and pharmacologic inhibition of METTL3 in HK-2 cells decreased TGF-β-induced fibrotic marker expression. STM2457-induced inhibition of METTL3 attenuated the degree of kidney fibrosis in vivo. Furthermore, METTL3 protein expression was significantly increased in the tissues of CKD patients with diabetic or IgA nephropathy. Therefore, targeting alterations in RNA methylation could be a potential therapeutic strategy for treating kidney fibrosis.

## RNA methylation: a new player in chronic kidney disease progression

Kidney fibrosis, a significant element in chronic kidney disease, is connected to RNA methylation (a process that controls gene activity). In research by Jung Pyo Lee and Sung-Yup Cho, they examined changes in RNA methylation during kidney fibrosis and its possibility as a treatment target. The scientists used both in vitro (in a controlled environment like a test tube) and in vivo (within a living organism) models of kidney fibrosis, discovering that stopping METTL3, a crucial enzyme in RNA methylation, lessened kidney fibrosis. They also observed a considerable increase in METTL3 protein expression in the tissues of patients with chronic kidney disease, implying that focusing on changes in RNA methylation could be a potential strategy for treating kidney fibrosis. This study emphasizes the significance of RNA methylation in kidney fibrosis and its potential as a treatment target.

This summary was initially drafted using artificial intelligence, then revised and fact-checked by the author.

## Introduction

Chronic kidney disease (CKD) is emerging as a major public health issue owing to an increase in overall prevalence and socioeconomic expenditure^1^. The overall prevalence of CKD is estimated to be 8–13%^2^. The overall prognosis of patients worsens with the progression of CKD, and some patients eventually progress to end-stage kidney disease that requires kidney replacement therapy, including dialysis or kidney transplantation. Importantly, disease-specific treatment for CKD is still lacking^3^. Among the various pathological mechanisms of CKD development and progression, kidney fibrosis has attracted attention as a therapeutic target for CKD^4^. Kidney fibrosis is characterized by the pathological accumulation of extracellular matrix proteins associated with the activation of myofibroblasts and involves various molecular signaling pathways, including TGF-β family cytokines, connective tissue growth factor, nuclear factor κB (NF-κB), Smad signaling, Wnt/β-catenin, Notch, and other growth factors^5^.

RNA methylation is a post-transcriptional RNA modification that regulates various intracellular signaling pathways and is involved in many critically important biological processes by regulating several aspects of RNA processing^6^. The most prevalent internal mRNA modification is N^6^-methyladenosine (m^6^A), which is deposited on mRNA by m^6^A methyltransferases (METTL3/14, WTAP, RBM15/15B, VIRMA, and ZC3H13, termed “writers”), removed by demethylases (FTO, ALKBH5, and ALKBH3, termed “erasers”), and recognized by m^6^A-binding proteins (YTHDC1/2, YTHDF1/2/3, IGF2BP1/2/3, HNRNP, and eIF3, termed “readers”). RNA methylation is associated with various inflammatory states, including autoimmunity, infection, metabolic diseases, cancer, neurodegenerative diseases, cardiovascular diseases, and bone diseases^7^. Recently, m^6^A RNA methylation was shown to be related to fibrosis in the lung, liver, and kidney^8–10^.

This study investigated the remodeling of the RNA methylation profile during kidney fibrosis in various kidney fibrosis models and the inhibitory effect of RNA methylation on CKD development and progression via specific inhibition of METTL3. Epitranscriptomic reprogramming of m^6^A RNA methylation associated with kidney fibrosis was explored using methylated RNA immunoprecipitation sequencing (MeRIP-seq). The expression of the m^6^A writer METTL3 was examined in human kidney tissues collected from CKD patients with IgA nephropathy and diabetic kidney disease. Our study suggests that remodeling of m^6^A RNA methylation during kidney fibrosis provides a therapeutic target for patients with CKD.

## Materials and methods

### Establishment of an in vitro model of kidney fibrosis

We established an in vitro TGF-β-induced fibrosis model using HK-2 cells. HK-2 cells (1 × 10^5^ cells/well) were seeded in six-well plates. We examined fibrosis-related epithelial-to-mesenchymal transition (EMT) and RNA methylation markers at different time points (0, 8, 24, and 48 h) with various concentrations of TGF-β (1, 10, and 100 ng/mL). In vitro experiments were performed at least twice to evaluate the statistical significance of the results.

### Animal models of UUO and kidney fibrosis

A two-week UUO mouse model was established and used in this experiment to establish kidney fibrosis. This study was approved by the Seoul National University Boramae Medical Center Institutional Animal Care and Use Committee (No. B22-0024-001). Animal experiments were carried out in the animal laboratory of the Seoul National University Boramae Medical Center under specific pathogen-free conditions in accordance with the National Research Council’s “Guidelines for the Care and Use of Laboratory Animals”. Male C57BL/6 mice (20–22 g, seven weeks old, Koatech, Kyeonggi-do, Korea) were given one week to adjust to the laboratory environment. Animals were randomly divided into two experimental groups/models (7-day and 14-day UUO, *n* = 7/experimental group, and *n* = 2 in the sham group for each experiment). Rompun^TM^ (xylazine 10 mg/kg; Bayer Korea Co., Ansan, Korea) and Zoletil^TM^ (zolazepam 30 mg/kg; Virbac Korea, Seoul, Korea) were intraperitoneally injected into the mice to induce anesthesia. The animals were placed on heating pads throughout the treatment to keep their temperature at ~37 °C. Following a left flank incision, the left kidney of the mouse was moved to the outside, exposing the kidney pedicles for manipulation. Proper adipose and connective tissue dissection was performed on the pedicle at the lower pole of the left kidney containing the ureter. The exposed pedicle was tied twice at the distal and proximal parts using 4-0 black silk to obstruct the urinary flow and induce hydronephrosis. The pedicle of the left kidney in the sham group was not ligated but was only exposed during the procedure. Mice were sacrificed on postoperative Day 7 or 14, and the left kidney was removed and prepared for the experiment. The left kidney was cut transversely into upper and lower halves. The upper half was divided transversely into three specimens, which were used for mRNA extraction, frozen tissue specimens, and western blot analyses to determine protein expression. The lower half was divided transversely into two pieces with higher paraffin tissue blocks for histological examination and the lower part for protein expression using western blotting. At least two independent animal experiments were conducted to assess statistical significance.

### Histological examination for fibrosis and immunohistochemistry

Kidney tissues were removed from the sacrificed mice and embedded in paraffin blocks. Embedded kidney tissues were sectioned into 4 μm thick slices and stained with periodic acid–Schiff, Masson’s trichrome, and Sirius red staining (all from ScyTek Laboratories, Logan, UT, USA) to evaluate the tissue morphology and kidney tissue fibrosis^11^. At least eight fields were randomly selected from each kidney segment and photographed under a light microscope (BX53F2; Olympus, Tokyo, Japan). The areas of fibrosis and total tissue were measured at ×100 magnification using ImageJ 1.52d software (Wayne Rasband, National Institute of Health, USA). The extent of fibrosis was assessed at ×100 magnification using ImageJ (v1.53e, Wayne Rasband, National Institute of Health, USA)^12,13^.

Paraffinized kidney tissue section (4 μm thick) were deparaffinized using a solution of xylene and rehydrated with ethanol. Antigen retrieval was performed by heating the sections three times in a microwave oven for 5 min, followed by immersion in 10% citrate buffer solution (pH 6.0). The sections were subsequently incubated with hydrogen peroxide in methanol (3%) for 10 min at room temperature to suppress endogenous peroxidase activity. Kidney tissue sections were incubated overnight at 4 °C with the primary METTL3 antibody (Cell Signaling Technology, Beverly, MA, USA; #86132). Rabbit primary antibodies were used with the Polink HRP DAB kit (GBI Labs, Bothell, WA, USA). Mayer’s hematoxylin was used as a counterstain (ScyTek Laboratories, Logan, UT, USA). At least five carefully chosen fields of the stained slides were imaged and examined at a magnification of ×200. The proportion of METTL3-positive regions was calculated using ImageJ (v1.53e; Wayne Rasband, National Institute of Health, USA)^12,13^.

### Western blot analysis

Protein was extracted from HK-2 cells, which were incubated with TGF-β for 48 h, and homogenized kidney tissues were collected two weeks after UUO induction using RIPA buffer containing a full protease inhibitor cocktail (Thermo Fisher, Rockford, IL, USA). Protein concentrations were determined using the bicinchoninic acid (BCA) test (Thermo Scientific, Rockford, IL, USA), and equivalent amounts of protein extracts were separated using 10% sodium dodecyl sulfate‒polyacrylamide gel electrophoresis. Proteins were subsequently transferred to a nitrocellulose membrane (Millipore Corporation, Bedford, MA, USA). Next, the membranes were blocked with 5% skim milk containing 2% BSA buffer and incubated with specific primary antibodies overnight at 4 °C (Supplementary Table 1). Anti-mouse or anti-rabbit IgG antibodies (Thermo Fisher Scientific, Rockford, IL, USA) were used as secondary antibodies. Protein bands were visualized using an improved chemiluminescence system (Advansta, CA, USA).

### Quantitative reverse transcription PCR (qRT‒PCR)

mRNA expression was evaluated using qRT‒PCR. Kidney cell lysates and total RNA were extracted using TRIzol reagent (Bioline, Luckenwalde, Germany). A Reverse Transcription System kit (Promega, Madison, WI, USA) was used to synthesize cDNA from 1 μg of RNA from each tissue sample. The expression levels of the METTL3, METTL14, METTL16, FTO, ALKBH5, YTHDF1, YTHDF2, and GAPDH genes were measured using quantitative RT‒PCR on a CFX96 Real-Time Detection System (Bio-Rad, Hercules, CA, USA) using each primer and assay-on-demand SYBR Green (Supplementary Table 2). Relative quantification was performed using the ΔΔCT method after the mRNA levels of various genes were normalized to those of GAPDH.

### Measurement of the m^6^A RNA methylation level

The levels of m^6^A were quantified using the EpiQuik m6A RNA Methylation Quantification Kit (Epigentek, Farmingdale, NY, USA) according to the manufacturer’s protocol^14^. Briefly, 80 mL of the binding solution was added to each well before use. Two microliters of negative control (NC), 2 μL of diluted positive control (PC), or 300 ng of sample RNA was then plated into the wells, and the solution was mixed by gently tilting from side to side. The strip plate was sealed and incubated at 37 °C for 90 min. Each well was washed three times with 150 μL of diluted wash buffer by pipetting. The diluted capture antibody (50 μL) was added to each well and incubated at room temperature for 60 min. The capture antibody solution was removed, and each well was washed three times with 150 μL of diluted wash buffer. Diluted detection antibodies (50 μL) were added to each well and incubated at room temperature for 30 min. Each well was washed four times with 150 μL of wash buffer, and 50 μL of enhancer solution was added to each well and incubated at room temperature for 30 min. Each well was washed five times with 150 μL of wash buffer. One hundred microliters of developer solution was added to each well and incubated at room temperature for 10 min in the dark. A stop solution (100 μL) was added to each well to stop the enzyme reaction, and the absorbance was measured on a microplate reader at 450 nm. The m^6^A quantity was calculated using the following formula:

$$m6A\, \% =\frac{(Sample\,OD-NC\,OD){\div}S}{(PC\,OD-NC\,OD){\div}P}\times 100 \%$$

where *S* is the amount of the input sample RNA (ng) and *P* is the amount of input positive control (PC) in nanograms.

### MeRIP-seq

MeRIP-seq and data analysis were performed as previously described with minor modifications^15^. Briefly, mRNA was purified from total RNA using a Dynabeads™ mRNA Purification Kit (Thermo Fisher, Rockford, IL, USA). Then, 5 μg of mRNA was fragmented and immunoprecipitated using an anti-m^6^A antibody (202003, Synaptic Systems, Gottingen, Germany); the immunoprecipitated RNA was washed and eluted by competition with N^6^-methyladenosine (Sigma-Aldrich, M2780). The purified RNA fragments from m^6^A MeRIP were used for library construction using the SMARTer Stranded RNA-seq kit (Clontech) and sequenced using an Illumina NovaSeq 6000 system.

The quality of the raw FASTQ files was assessed using FastQC (v0.11.9)^16^. The reads were trimmed using Trimmomatic (v 0.39)^17^ and in-house methods. Trimmed reads were aligned to the human (GRCh38) or mouse (mm10) reference genome using STAR (v2.7.8a)^18^, and gene expression levels were quantified using RSEM (v1.3.3)^19^. m^6^A peaks and DMPs were detected using the exomePeak2 R package (v1.3.7)^20^. Peaks were annotated using an in-house method using the gencode.v22.annotation.gtf file. Significant DMPs were considered those with [fold change] >3/2 or [fold change] <2/3 and *P* value < 0.05. The m^6^A peak distribution was visualized using the Guitar R package (v2.6.0)^21^. Motif discovery was performed using findMotifsGenome.pl from HOMER (v4.11)^22^. Volcano plots were generated using the EnhancedVolcano R package (v1.12.0)^23^, and the peak of interest was visualized using IGV software (v2.12.2). Analysis of sequencing data was mostly carried out using the computing server at the Genomic Medicine Institute Research Service Center.

### MeRIP-qPCR and RNA immunoprecipitation (RIP)-qPCR

RNA was fragmented by repeated heating at 94 °C for 2 min and vortexing. The fragmented RNA was purified using RNA Clean and Concentrator kits (Zymo Research, Irvine, CA, USA). Protein A/G Magnetic Beads (Pierce, 88802) were used for immunoprecipitation with 1 μg of anti-m^6^A antibody (Synaptic Systems, 202003) and 20 μg of total RNA for each reaction. Two micrograms of total RNA was preserved as RNA input. Immunoprecipitation tubes were incubated overnight at 4 °C. The beads were washed, and RNA was eluted and purified using an RNA Clean & Concentrator kit. RNA levels were analyzed using quantitative RT‒PCR and primers for *NET1*: 5’-CGCTTACAGATGTGGCTCTG-3’ and 5’-ATGATGCTCCCCTTACGAGA-3’. For RIP-qPCR, cell lysates were immunoprecipitated with an anti-IGF2BP1 antibody, and subsequently, the recovered RNAs were subjected to quantitative RT‒PCR using primers for *NET1*.

### Suppression of METTL3 expression using siRNA transfection or STM2457

To inhibit the production of RNA methylation-related proteins, we transfected HK-2 cells with siRNAs for METTL3, FTO, and YTHDF1 (sequence provided in Supplementary Table 3). HK-2 cells (1 × 10^5^ cells/well) were grown in antibiotic-free growth medium with fetal bovine serum and incubated in a CO_2_ incubator at 37 °C for one day prior to transfection. For cell transfection, a mixture of Transfection Reagent (siRNA duplex solution mixed with Transfection Medium, both acquired from Invitrogen) was used.

In this study, we used STM2457 (Chemicals, DC53045, Shanghai, China), a selective inhibitor of METTL3 catalytic activity, as a pharmacological inhibitor of METTL3 in in vitro and in vivo experiments. HK-2 cells were challenged with TGF-β (10 ng/mL for 48 h), and STM2457 was added at different doses (1, 5, and 10 μM). In the UUO experiment, mice were randomly allocated to four experimental groups (sham/vehicle, *n* = 4; sham/STM2457, *n* = 4; 14-day UUO/vehicle, *n* = 7; 14-day UUO/STM2457, *n* = 6). STM2457 was dissolved in a 2-hydroxypropyl cyclodextrin vehicle at a concentration of 20% (w/v) (Sigma, H107)^24^. The mice were administered vehicle or STM2457 (50 mg/kg) daily for 2 weeks.

### Measurement of METTL3 expression in tissues obtained from patients with CKD

We enrolled 16 patients with CKD, of which eight had diabetic nephropathy, eight had IgA nephropathy confirmed by kidney biopsy, and 11 were control patients. A kidney biopsy was performed prospectively to evaluate the primary cause of kidney disease, and participants signed a voluntary informed consent form before enrollment. This prospective study was approved by the Institutional Review Board of the Seoul National University Boramae Medical Center (No. 06-2011-50). Patient baseline demographic and clinical information, including age, sex, serum creatinine, estimated glomerular filtration rate levels, urinary albumin-to-creatinine ratio, and pathological reports of kidney biopsy, were obtained from electronic medical records.

### Statistical analysis

Data are presented as the mean and standard deviation. Standard deviations and mean values are shown in bar graphs, and individual data are depicted as dot plots. Statistical analyses were performed using Prism 9.4.0 (GraphPad Software, San Diego, CA, USA) and SPSS (version 24.0; IBM Corp., Amrok, NY, USA). Differences between groups were examined using an independent *t* test or one-way ANOVA with a post hoc Tukey test, as appropriate. A *P* value of 0.05 was used as the cutoff point for statistical significance.

## Results

### Alterations in the levels of m^6^A RNA methylation-associated genes in the TGF-β-induced cell fibrosis model

**Fig. 1:**
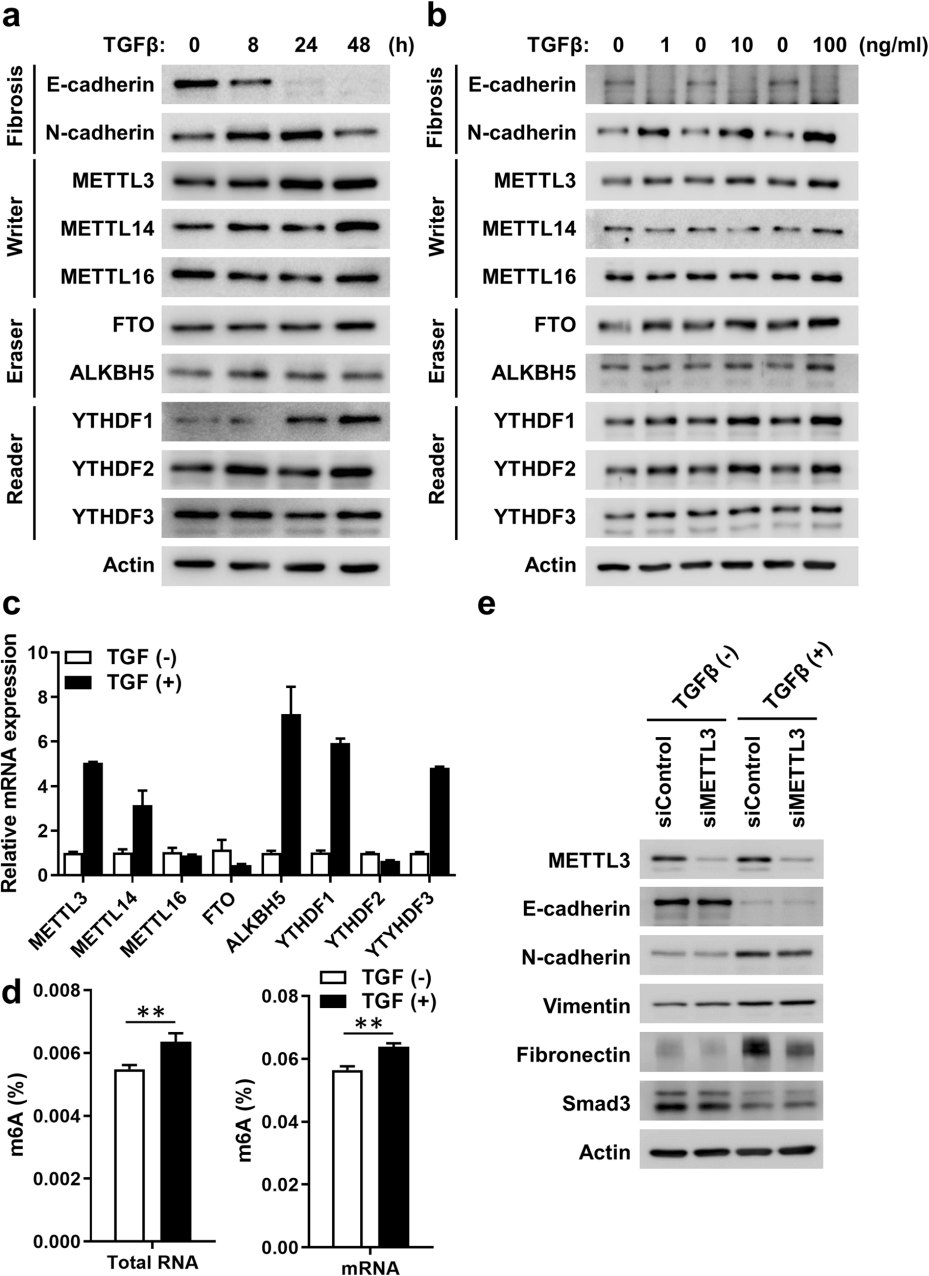
TGF-β increased N^6^-methyladenosine (m^6^A) RNA methylation in renal tubular epithelial cells. **a** Western blot of HK-2 cells for fibrosis-related and m^6^A RNA methylation writer, eraser, and reader proteins after TGF-β challenge (10 ng/mL) at baseline and at 8, 24, and 48 h. **b** Western blot of fibrosis-related and RNA methylation proteins in HK-2 cells with TGF-β challenge (1, 10, and 100 ng/mL) for 48 h. **c** Messenger RNA levels of RNA methylation-related markers with or without TGF-β challenge (10 ng/mL for 48 h) analyzed using real-time PCR. **d** m^6^A RNA methylation levels after TGF-β challenge (10 ng/mL for 48 h) estimated using enzyme-linked immunosorbent assays for RNA m^6^A. An independent unpaired *t* test was used for comparisons between two groups. ^**^P < 0.01. **e** Effect of METTL3 knockdown on TGF-β-mediated gene expression of fibrosis-related genes. After METTL3 was inhibited using siMETTL3, HK-2 cells were challenged with TGF-β (10 ng/mL for 48 h).

The TGF-β-challenged human kidney tubular epithelial cell (HK-2) fibrosis model was used to evaluate alterations in the RNA methylation profile during kidney fibrosis. The levels of fibrosis-related and RNA methylation writer, eraser, and reader proteins after TGF-β challenge (10 ng/mL) at baseline and 8, 24, and 48 h are shown in the western blot results in Fig. 1a. The levels of N-cadherin increased as those of E-cadherin, a representative marker of EMT, decreased over time. RNA methylation-related METTL3, FTO, and YTHDF1 expression was also increased after TGF-β administration. An increase in the levels of METTL3, FTO, and YTHDF1 was also observed at all tested doses of TGF-β (Fig. 1b, 1, 10, and 100 ng/mL). The RNA expression levels of RNA methylation-related proteins are shown in Fig. 1c. METTL3, METTL14, ALKBH5, YTHDF1, and YTHDF3 mRNA expression significantly increased after TGF-β challenge. Furthermore, the amount of m^6^A RNA methylation after TGF-β treatment significantly increased (Fig. 1d). The inhibitory effects of METTL3, an RNA methylation writer, were tested using an siRNA against METTL3. METTL3 expression was successfully inhibited after siRNA treatment, and the TGF-β-induced increase in N-cadherin and fibronectin expression was suppressed after knockdown of METTL3 (Fig. 1e). We also evaluated the inhibitory effects of FTO and YTHDF1 using siRNAs (Supplementary Fig. 1). Knockdown of FTO increased the levels of one of the fibrotic markers, fibronectin, in the presence of TGF-β compared to those of the control cells (Supplementary Fig. 1a), but YTHDF1 inhibition had a negligible effect on fibrotic markers, in contrast to METTL3 inhibition (Supplementary Fig. 1b).

Among m^6^A reader proteins, IGF2BPs (IGF2BP1, IGF2BP2, and IGF2BP3) have been reported to promote the stability of target mRNAs^6^. When we treated HK-2 cells with TGF-β, we found that TGF-β increased IGF2BP1 expression, while TGF-β slightly decreased the expression levels of IGF2BP2 and IGF2BP3 (Supplementary Fig. 2a, b). When we knocked down IGF2BPs, we found that depletion of IGF2BP1 decreased the expression of EMT markers, including N-cadherin, vimentin, and fibronectin, in the presence of TGF-β, but knockdown of IGF2BP2 and IGF2BP3 had little effect on the TGF-β-induced expression of EMT markers (Supplementary Fig. 2c–e). These data suggested that IGF2BP1 plays roles in the TGF-β-induced EMT process as one of the main reader proteins.

### Alterations in the levels of m^6^A RNA methylation-associated genes in the unilateral ureteral obstruction (UUO) mouse model of kidney fibrosis

**Fig. 2:**
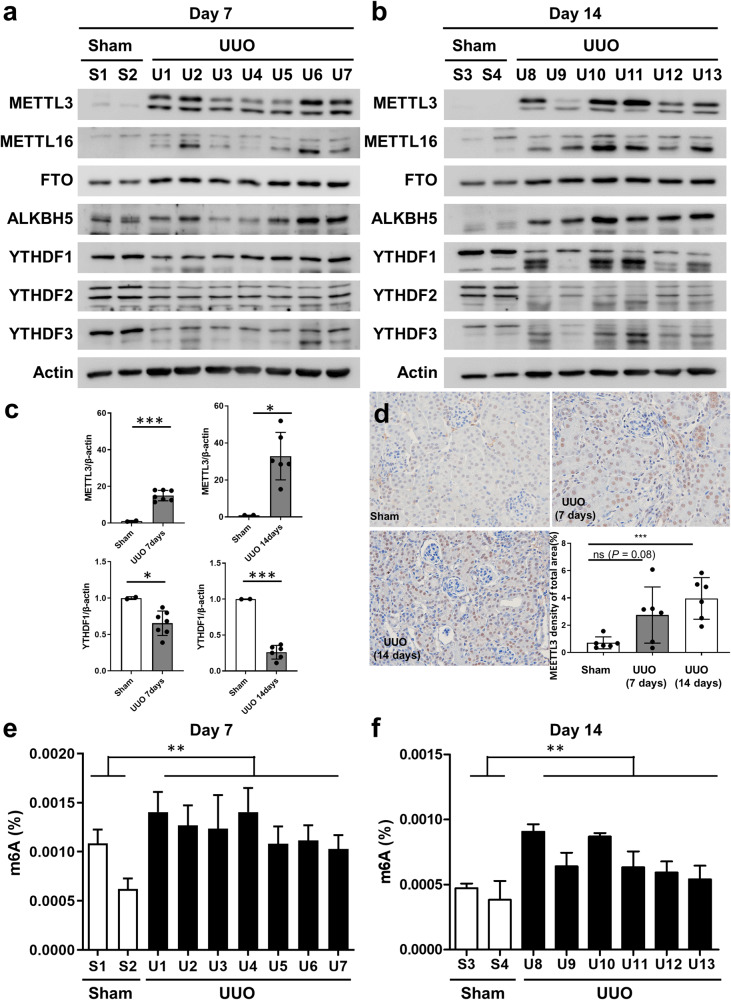
The levels of N^6^-methyladenosine (m^6^A) RNA methylation increased in a unilateral ureteral obstruction (UUO) mouse model. **a**, **b** Western blot for fibrosis-related and m^6^A RNA methylation writer, eraser, and reader proteins in 7-day (a) and 14-day (b) UUO-induced mouse models. **c** Densitometric measurement of western blots for METTL3 and YTHDF1 in 7-day and 14-day UUO-induced mouse models. **d** Immunohistochemical staining for METTL3 in 7-day and 14-day UUO-induced mouse models. **e**, **f** m^6^A RNA methylation levels in 7-day (e) and 14-day (f) UUO-induced mouse models estimated using an enzyme-linked immunosorbent assay for RNA m^6^A. Comparisons of means between two and three groups were analyzed using independent *t* tests and ANOVA with post hoc Tukey tests, respectively. ^ns^*P* > 0.05, ^*^P < 0.05, ^**^P < 0.01, ^***^P < 0.001.

UUO-treated mouse kidneys showed increased tissue fibrosis, and the difference was significant in the 14-day UUO model (Supplementary Fig. 3). The levels of fibrosis-related and RNA methylation writer, eraser, and reader proteins in the 7-day and 14-day UUO mouse models are shown in the western blot results in Fig. 2a, b, respectively. The protein expression of METTL3 was significantly increased, while that of YTHDF1 was decreased in both the 7-day and 14-day UUO models (Fig. 2c). The levels of METTL16, FTO, and ALKBH5 showed an increasing trend, whereas those of YTHDF2 and YTHDF3 showed a decreasing trend (Fig. 2a, b). The kidney tissue area with METTL3 protein expression was significantly increased in the 14-day UUO mouse model (Fig. 2d). The total levels of m^6^A RNA methylation significantly increased in both the 7-day and 14-day UUO models (Fig. 2e, f).

### Effect of METTL3 knockdown on transcriptomic remodeling in TGF-β-challenged HK-2 cells

**Fig. 3:**
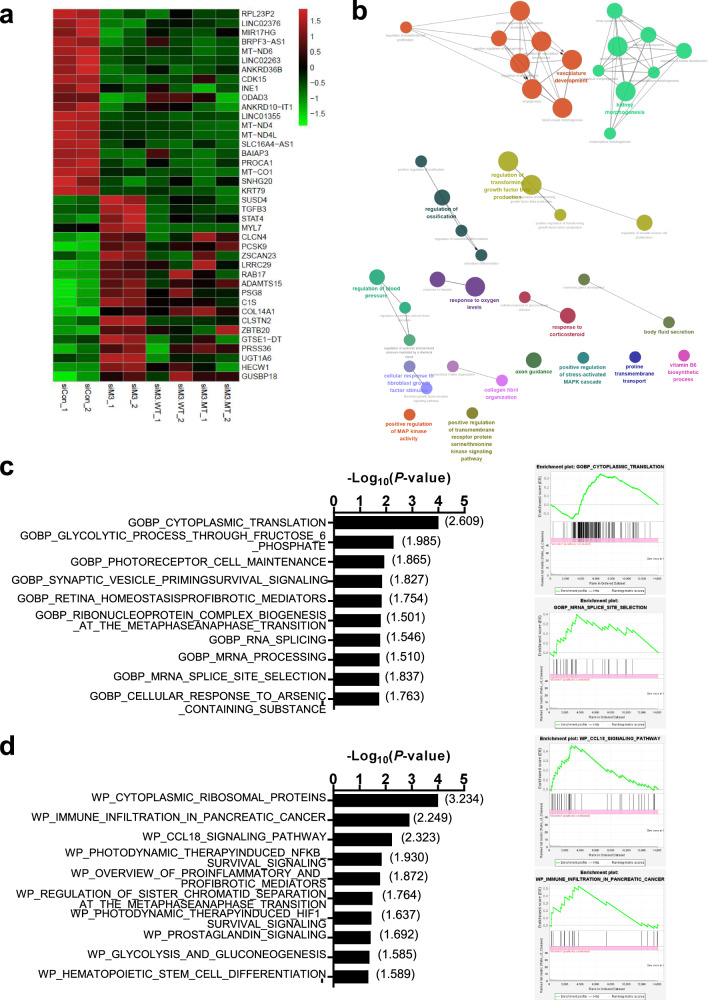
Transcriptomic effect of METTL3 knockdown on TGF-β-mediated epithelial-mesenchymal transition in renal tubular epithelial cells. **a** Top 40 differentially expressed genes following METTL3 knockdown in TGF-β-treated HK-2 cells. After transfection of siRNA targeting the 3’ UTR of METTL3 and METTL3-overexpressing vectors resistant to siRNA, HK-2 cells were treated with TGF-β (10 ng/mL for 48 h), and their transcriptome was analyzed using RNA sequencing. Heatmap demonstrating the top 40 differentially expressed genes (*P* < 0.05, |fold change| ≥1.5) sorted by fold change. Each row represents a gene, and each column represents a sample. Red and green indicate expression levels above and below the median of each gene across the samples, respectively. **b** Network representation of enriched Gene Ontology (GO) biological processes (analyzed by ClueGO; *P* < 0.05) using 372 differentially expressed genes following treatment with METTL3 siRNA (*P* < 0.05, |fold change| ≥1.5). The node size represents the term enrichment significance. The nodes are linked based on their kappa score ( ≥ 0.4), where the term labels with the most genes per group are shown. **c** Gene set enrichment analysis (GSEA) for Gene Ontology biological process (GOBP) gene sets using RNA sequencing data for TGF-β-treated (10 ng/mL for 48 h) HK-2 cells with the control and METTL3 siRNAs. The left panel shows the enriched GOBP gene sets (*P* < 0.02) in the control siRNA-treated cells compared with the METTL3 siRNA-treated cells. The right panel shows the enrichment plots for representative GOBP gene sets. On the *x* axis, the genes are ranked from the most upregulated to the most downregulated between the control siRNA-treated cells (left end; positively correlated) and the METTL3 siRNA-treated cells (right end; negatively correlated). The *y* axis shows a running enrichment score for METTL3 siRNA treatment. **d** GSEA for WikiPathways gene sets using RNA sequencing data for the TGF-β-treated (10 ng/mL for 48 h) HK-2 cells with the control and METTL3 siRNAs. The left panel shows the enriched WikiPathways gene sets (*P* < 0.05) in the control siRNA-treated cells compared with the METTL3 siRNA-treated cells. The right panel shows the enrichment plots for representative WikiPathways gene sets. On the *x* axis, the genes are ranked from the most upregulated to the most downregulated between the control siRNA-treated cells (left end; positively correlated) and the METTL3 siRNA-treated cells (right end; negatively correlated). The *y* axis shows a running enrichment score for METTL3 siRNA treatment.

Because changes in the m^6^A RNA methylation profile are highly associated with transcriptomic remodeling of mRNAs, we investigated the effect of *METTL3* knockdown on transcriptomic alterations in the TGF-β-challenged HK-2 cells. When the expression of *METTL3* was downregulated by siRNAs in TGF-β-challenged HK-2 cells, 219 genes were significantly upregulated by treatment with siRNA against *METTL3*, and 153 genes were significantly downregulated (*P* < 0.05, |fold change| ≥ 1.5; Fig. 3a and Supplementary Fig. 4a, b). Analysis of the top 40 differentially expressed genes following METTL3 knockdown revealed that in 29 of 40 genes, the effects of siRNA tended to be reversed by METTL3 overexpression, and approximately half of these genes exhibited a dependency on METTL3 activity (Fig. 3a). These genes contain the canonical motifs for m^6^A modification. Gene Ontology (GO) analysis using ClueGO^25^ showed that these 372 differentially expressed genes were enriched in several EMT-related GO terms, such as ‘regulation of transforming growth factor beta production’, ‘positive regulation of transmembrane receptor protein serine/threonine kinase signaling pathway’, and ‘collagen fibril organization’, and morphogenesis-related GO terms, including ‘kidney morphogenesis’ and ‘vasculature development’ (*P* < 0.05; Fig. 3b). When the RNA sequencing data were used in gene set enrichment analysis (GSEA)^26^ with Gene Ontology biological process gene sets, several gene sets associated with RNA processing, including ‘cytoplasmic translation’, ‘RNA splicing’, ‘mRNA processing’, and ‘mRNA splice site selection’, were enriched in the control cells compared to the *METTL3* knockdown cells (*P* < 0.02; Fig. 3c), suggesting that *METTL3* knockdown suppressed these pathways. In addition, GSEA using WikiPathways (https://www.wikipathways.org/) gene sets showed enrichment of inflammation-related gene sets, such as ‘immune infiltration in pancreatic cancer’, ‘CCL18 signaling pathway’, and ‘overview of proinflammatory and profibrotic mediators’, in the control cells compared to the *METTL3* knockdown cells (*P* < 0.05; Fig. 3d).

### TGF-β-induced alterations in the m^6^A profiles of HK-2 cells

**Fig. 4:**
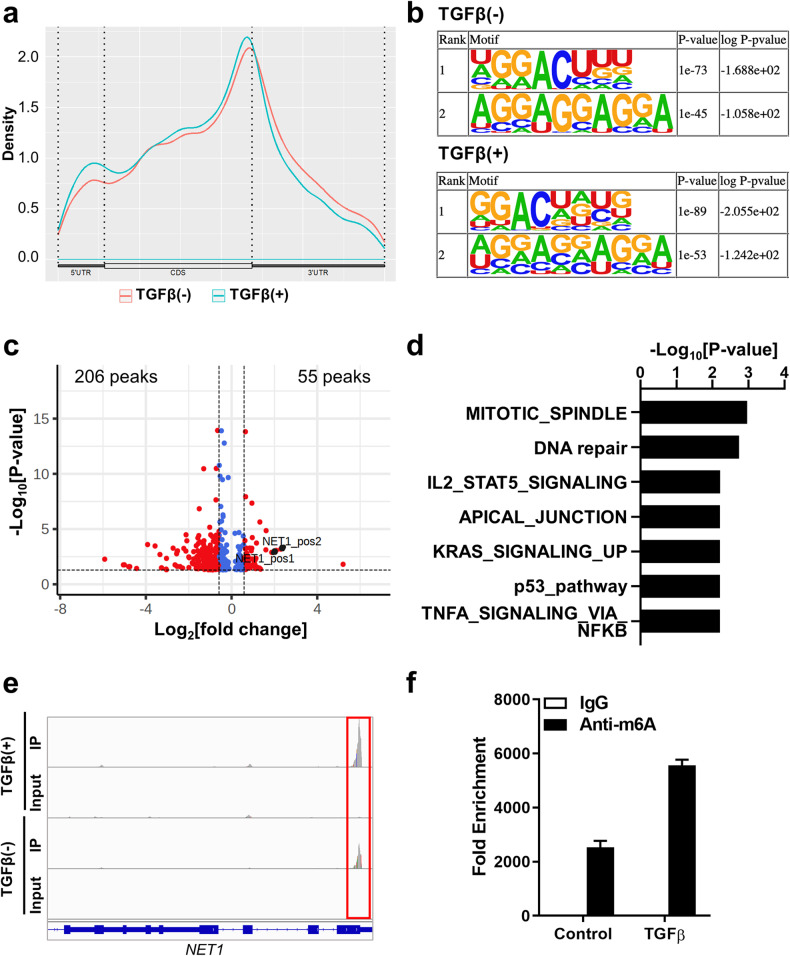
Profile of N^6^-methyladenosine (m^6^A) RNA methylation in in vitro kidney fibrosis models. **a** M^6^A peak distribution across the mRNA transcripts, including 5′ untranslated regions (5′ UTR), coding DNA sequence (CDS), and 3′ UTR. The RNA m^6^A profile was analyzed using methylated RNA immunoprecipitation sequencing (MeRIP-seq). **b** Enriched consensus m^6^A motifs were identified from the MeRIP-Seq analysis in the control and TGF-β-treated (10 ng/mL for 48 h) HK-2 cells. **c** Volcano plot for differentially methylated peaks in transcript levels from the MeRIP-Seq analysis between the control and TGF-β-treated HK-2 cells ([fold change] >3/2 or <2/3, *P* < 0.05). The *x* axis represents fold changes in the TGF-β-treated cells compared with the control cells. **d** Significantly enriched hallmark gene sets (*P* < 0.01) in 261 genes with differentially methylated m6A peaks between the control and TGF-β-treated HK-2 cells. **e** The Integrative Genomics Viewer (IGV) tool revealed the m^6^A peak distribution in *NET1* from the MeRIP-Seq analysis in the control (TGF-β [-]) and TGF-β-treated (TGF-β [+]) HK-2 cells. **f** M^6^A methylation levels of the *NET1* transcript in the control and TGF-β-treated HK-2 cells estimated using MeRIP-qPCR.

As TGF-β treatment increased METTL3 expression and total cellular m^6^A levels in HK-2 cells (Fig. 1a, d), we analyzed the TGF-β-induced alterations in m^6^A profiles in mRNAs by performing MeRIP-seq (Supplementary Fig. 5). In accordance with previous studies, m^6^As were enriched around the stop codon of the mRNAs (Fig. 4a), and the GGAC sequence was the most enriched transcript sequence with or without TGF-β treatment (Fig. 4b). We detected 9815 peaks in the TGF-β-treated HK-2 cells and 7011 peaks in the untreated cells, suggesting that mRNAs of 5360 genes were subjected to m6A modifications in one or more of the two conditions. Among 372 differentially expressed genes by *METTL3* knockdown (Fig. 3a, b), 91 genes (24.6%) were detected in MeRIP-seq analysis of the TGF-β-treated or nontreated HK-2 cells, indicating modification by METTL3. When we analyzed the differentially methylated peaks (DMPs) in mRNAs, we found that a total of 55 peaks were increased (fold change >1.5, *P* < 0.05) and 206 were decreased (fold change <0.67 and *P* < 0.05) after TGF-β treatment (Fig. 4c). Gene set analysis using these 261 genes with DMPs demonstrated the enrichment of immune-related gene sets, including ‘IL2 STAT5 signaling’ and ‘TNFa signaling via NFkB’, and DNA damage-related gene sets, such as ‘DNA repair’ and ‘p53 pathway’ (*P* < 0.01; Fig. 4d and Supplementary Fig. 6).

Among the genes with increased methylated peaks following TGF-β treatment, we further analyzed the m^6^A methylation of the *NET1* (neuroepithelial cell transforming 1) gene because *NET1* has been suggested to play roles in TGF-β-induced EMT in human retinal pigment epithelial cells and mouse fibroblasts^27,28^. The MeRIP-seq data showed that TGF-β increased the m^6^A methylation of the *NET1* transcript around the transcription termination site (Fig. 4e). TGF-β-induced augmentation of m^6^A levels in *NET1* cells was validated using MeRIP-qPCR (Fig. 4f). In addition, we verified the interaction between *NET1* mRNA and the m^6^A reader IGF2BP1 through RIP-qPCR analysis (Supplementary Fig. 2f).

### NET1 as a possible regulator in TGF-β-induced fibrosis of kidney tubular epithelial cells

**Fig. 5:**
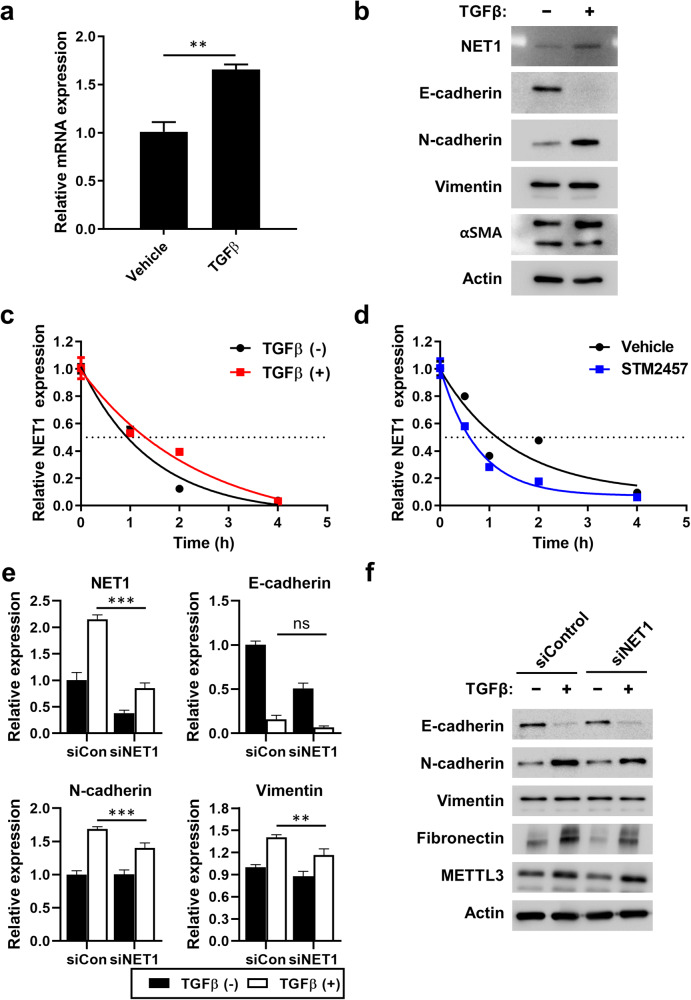
NET1 regulates TGF-β-induced epithelial-to-mesenchymal transition (EMT). **a**, **b** mRNA (**a**) and protein (**b**) levels of NET1 after TGF-β treatment (10 ng/mL for 48 h) estimated using real-time PCR and western blot, respectively. **c**, **d** RNA stability of *NET1* after treatment with TGF-β or the METTL3 inhibitor STM2457. Effect of TGF-β (**c**) or STM2457 (**d**) on *NET1* RNA stability evaluated by actinomycin D treatment (5 μg/mL) and real-time PCR for *NET1* transcript at the indicated times. **e**, **f** The effect of NET1 on TGF-β-induced EMT. Effects of NET1 knockdown using siRNA on the expression of EMT markers after TGF-β treatment (10 ng/mL for 48 h), as evaluated using real-time PCR (**e**) and western blot analysis (**f**).

Next, we investigated the role of NET1 in TGF-β-induced fibrosis of kidney tubular epithelial cells. TGF-β treatment increased the mRNA and protein expression of NET1 in HK-2 cells (Fig. 5a, b). Knockdown of *METTL3* resulted in a decrease in *NET1* mRNA levels, and overexpression of siRNA-resistant *METTL3* restored *NET1* mRNA levels (Supplementary Fig. 7). When we estimated the mRNA stability of *NET1* in the presence of actinomycin D, we found that TGF-β treatment enhanced the stability of *NET1* mRNA (Fig. 5c), probably due to increased m^6^A levels of *NET1* mRNA (Fig. 4f). In contrast, addition of the METTL3-specific inhibitor STM2457^24^ decreased the stability of *NET1* mRNA (Fig. 5d), suggesting that m^6^A modification of *NET1* mRNA is needed for the TGF-β-mediated stabilization of *NET1* mRNA.

When the expression of NET1 was downregulated by siRNA treatment, the TGF-β-increased mRNA expression of EMT markers, such as N-cadherin and vimentin, was suppressed compared to that in the control siRNA-treated cells (Fig. 5e). The protein levels of N-cadherin and fibronectin, which were induced by TGF-β treatment, were diminished by treatment with siRNA against *NET1* (Fig. 5f). In addition, overexpression of *NET1* enhanced TGF-β-induced expression of N-cadherin and vimentin in the *METTL3* knockdown HK-2 cells (Supplementary Fig. 8a, b). These data suggest that the TGF-β-induced stabilization and increase in *NET1* mRNA levels play a role in the TGF-β-mediated expression of EMT markers.

To investigate the effects of *NET1* knockdown on TGF-β-induced signaling in HK-2 cells, we performed RNA sequencing on control cells, TGF-β-treated cells, and TGF-β-treated cells with *NET1* knockdown. GSEA using KEGG pathways revealed significant enrichment of 15 gene sets in the TGF-β-treated cells compared to the controls (*P* < 0.05, Supplementary Fig. 9a). In the TGF-β-treated cells with *NET1* knockdown, 3 of these 15 gene sets showed significant depletion (*P* < 0.1; Supplementary Fig. 9b). Furthermore, 11 of the 15 gene sets exhibited a trend toward depletion in the TGF-β-treated *NET1* knockdown cells compared to the TGF-β-treated cells (normalized enrichment score >1 in TGF-β-treated cells; Supplementary Fig. 9b). EMT-related gene sets, such as ‘focal adhesion’, ‘adherent junction’, and ‘vascular smooth muscle contraction’, were included in these gene sets. These findings collectively suggest that *NET1* depletion leads to a reversal of several TGF-β-induced transcriptomic changes.

### Alterations in m^6^A profiles in the UUO model

**Fig. 6:**
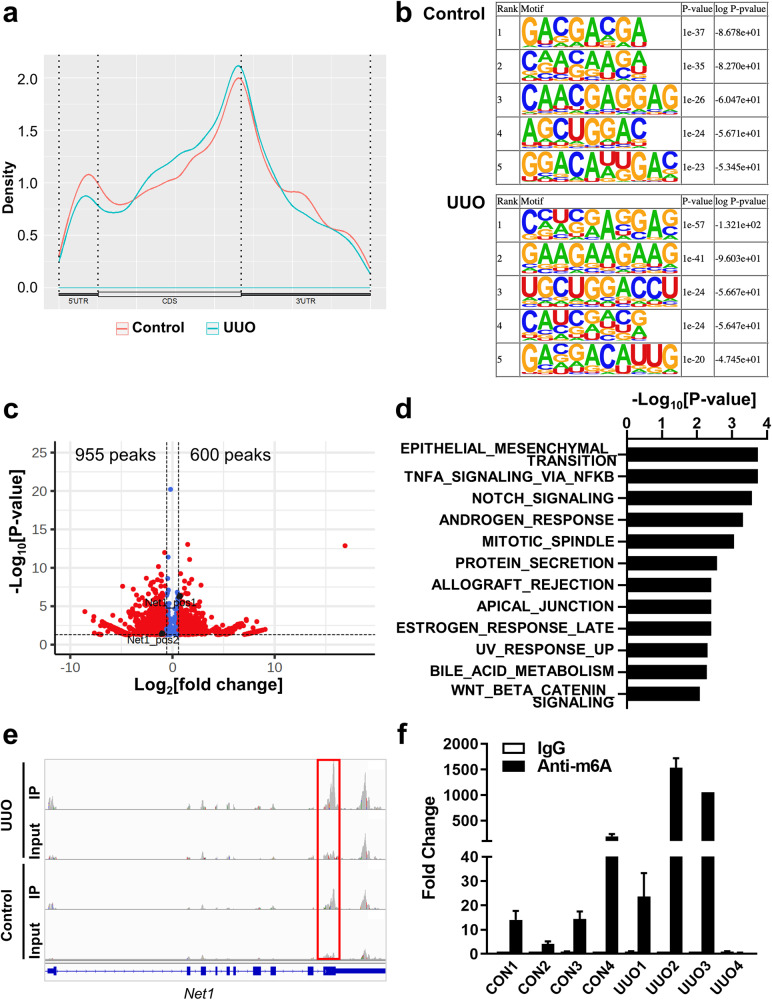
Profile of N^6^-methyladenosine (m^6^A) RNA methylation in in vivo kidney fibrosis models. **a** M^6^A peak distribution across the mRNA transcripts, including 5′ untranslated regions (5′ UTR), coding DNA sequence (CDS), and 3′ UTR. The RNA m^6^A profile was analyzed using methylated RNA immunoprecipitation sequencing (MeRIP-seq) using kidney tissues from control and unilateral ureteral obstruction (UUO)-induced model mice. **b** Enriched consensus m^6^A motifs were identified from the MeRIP-Seq analysis in the control and UUO-induced mice. **c** Volcano plot for differentially methylated peaks in transcript levels from the MeRIP-seq analysis between the control and UUO-induced mice ([fold change] >3/2 or <2/3, *P* < 0.05). The *x* axis represents fold changes in the UUO-induced mice compared with the control mice. **d** Significantly enriched hallmark gene sets (*P* < 0.01) in 472 genes with increased m^6^A peaks (fold change >2) in the UUO-induced mice compared with the control mice. **e** The Integrative Genomics Viewer (IGV) tool revealed the m^6^A peak distribution in the *NET1* gene from the MeRIP-Seq analysis in the control and UUO-induced mice. **f** M^6^A methylation levels of the *NET1* transcript in the control and UUO model mice estimated using MeRIP-qPCR.

Next, we analyzed in vivo alterations in mRNA m^6^A profiles in the UUO mouse model using MeRIP-seq. In accordance with the cell line data (Fig. 4a, b), m^6^As were enriched around the stop codon of the mRNAs (Fig. 6a), and the GGAC and CGCA sequences were enriched in transcript sequences in both the control and UUO mice (Fig. 6b). When we analyzed the DMPs in mRNAs, a total of 600 peaks were increased (fold change >1.5, *P* < 0.05), and 955 were decreased (fold change <0.67 and *P* < 0.05) in the UUO model mice (Fig. 6c). Gene set analysis using these 472 genes with increased DMPs (fold change >2) demonstrated the enrichment of EMT-related gene sets, including ‘epithelial-mesenchymal transition’, ‘notch signaling’, and ‘Wnt beta catenin signaling’, and immune-related gene sets, such as ‘TNFa signaling via NFkB’ and ‘allograft rejection’ (*P* < 0.01; Fig. 6d). Although there was individual variation, the m^6^A methylation levels of *NET1* were augmented in the UUO-treated mice compared to the control mice in the MeRIP-seq and MeRIP-qPCR experiments (Fig. 6e, f).

### Effects of METTL3 inhibition on fibrosis in vitro and in vivo

**Fig. 7:**
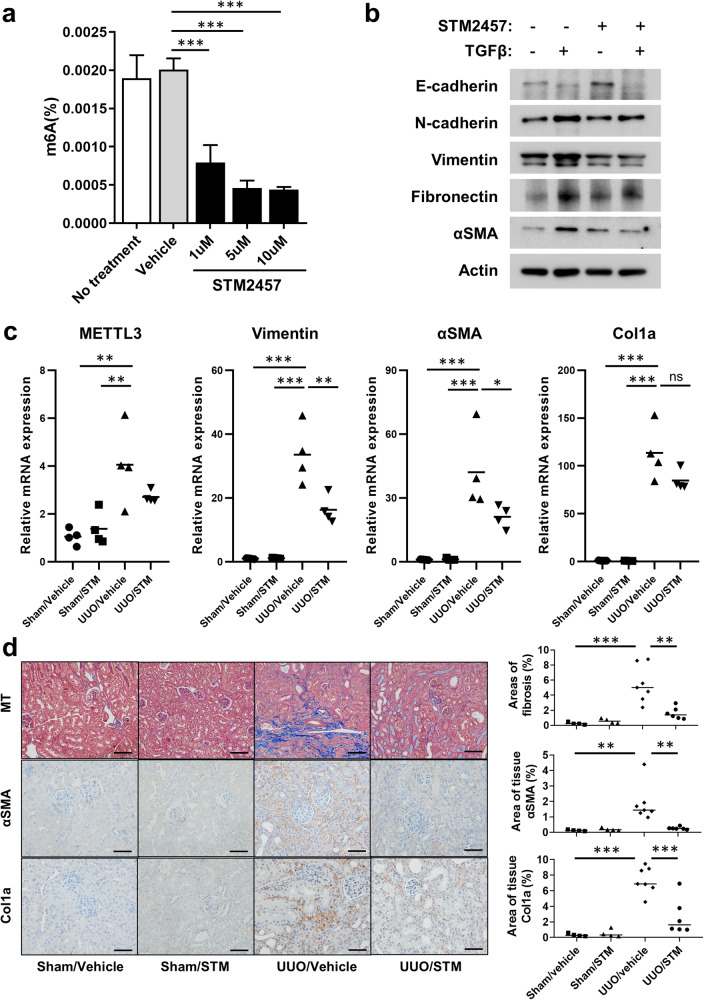
Effects of METTL3 inhibition on kidney fibrosis models. **a** Total RNA N^6^-methyladenosine (m^6^A) levels in HK-2 cells after treatment with the METTL3 inhibitor STM2457 (1, 5, and 10 μM for 24 h). **b** Western blot of fibrosis-related proteins in the HK-2 cells challenged with TGF-β (10 ng/mL for 48 h) with or without STM2457 (5 μM for 48 h). **c** RT‒PCR results for METTL3 and other fibrosis-related proteins in the 14-day UUO-induced mouse model. **d** Masson’s trichome stain (MT) and immunohistochemical staining results in the 14-day UUO-induced mouse model after treatment with STM2457 (50 mg/kg, daily). Comparisons of means between two and three or more groups were analyzed using independent *t* tests and ANOVA with post hoc Tukey tests, respectively. Scale bar: MT 100 μm (×200), αSMA/Col1a 50 μm (×400). ^ns^*P* > 0.05, ^*^P < 0.05, ^**^P < 0.01, ^***^P < 0.001.

Next, we investigated the effects of a METTL3-specific inhibitor in HK-2 cells challenged with TGF-β in an in vitro fibrosis model and a 14-day UUO in vivo mouse model. The change in m^6^A RNA methylation levels in HK-2 cells after treatment with the METTL3-specific inhibitor STM2457^24^ at different concentrations (1, 5, and 10 μM) is shown in Fig. 7a. The amount of m^6^A RNA methylation significantly decreased after treatment with STM2457 at all the tested concentrations. Higher doses of STM2457 were associated with greater inhibitory effects on cellular m^6^A levels, but the difference was not statistically significant. Protein expression levels during EMT and fibrosis are shown in Fig. 7b. The expression of vimentin, fibronectin, and α-SMA was suppressed after treatment with STM2457 in the presence of TGF-β. However, STM2457 exhibited a minimal effect on the TGFβ-induced expression of EMT markers in the *METTL3* knockdown cells (Supplementary Fig. 10), suggesting that the effect of STM2457 is mediated through the inhibition of METTL3. In addition, treatment with STM2457 decreased the cellular levels of m^6^A in rat kidney fibroblasts and NRK49F cells (Supplementary Fig. 11a, b). Furthermore, STM2457 suppressed the expression of all EMT markers in the presence of TGF-β in NRK49F cells (Supplementary Fig. 11a, c), suggesting that the METTL3 inhibitor shows antifibrotic effects even in kidney fibroblasts.

Figure 7c shows the mRNA expression levels of EMT and fibrotic markers in the UUO-treated mice with or without treatment with STM2457. The expression of vimentin and αSMA significantly decreased after treatment with STM2457 in the UUO models (Fig. 7c). Kidney fibrotic areas were measured with Masson’s trichrome staining, and kidney tissue expression of αSMA and Col1a was measured by immunohistochemical staining (Fig. 7d). The increased fibrotic area and expression of αSMA and Col1a were significantly decreased after STM2457 treatment in the UUO models.

### METTL3 expression in human CKD tissue

**Fig. 8:**
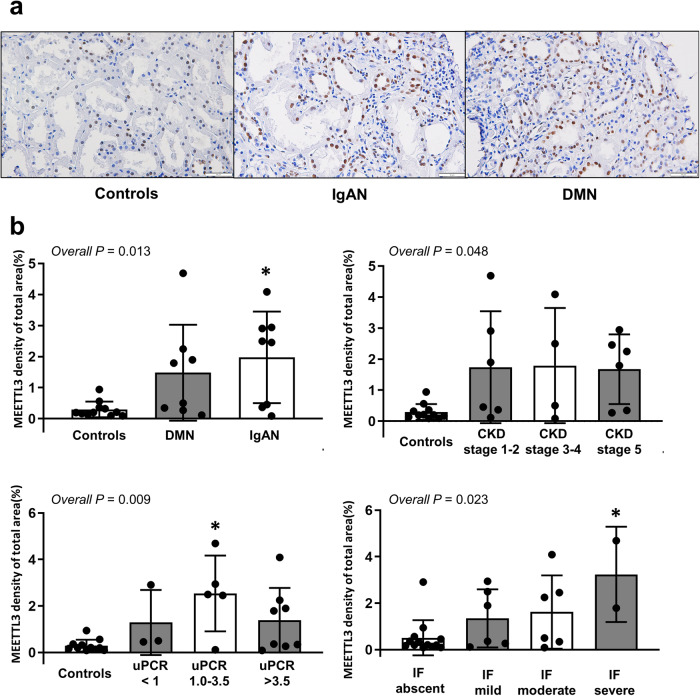
Tissue expression of METTL3 in patients with chronic kidney disease (CKD). **a** Representative METTL3 immunohistochemical staining results in kidney tissues from CKD patients with IgA nephropathy and diabetic nephropathy and control patients. Scale bar: 50 μm (×400). **b** Measurement of the METTL3-positive area according to disease type, CKD stage, proteinuria levels, and degrees of interstitial fibrosis. DMN diabetic nephropathy, IgAN IgA nephropathy, IF interstitial fibrosis, uPCR urinary protein-to-creatinine ratio (g/g). Comparisons of means among three or more groups were performed using an independent ANOVA with a post hoc Tukey’s test. ^*^P < 0.05.

Kidney tissue was collected from 16 CKD patients with diabetic nephropathy (*n* = 8) or IgA nephropathy (*n* = 8) and control subjects (*n* = 11). METTL3 expression in kidney tissues was determined using immunohistochemical staining. Figure 8a shows representative METTL3 immunohistochemical staining results for the three samples (IgA nephropathy, diabetic nephropathy, and control; scale bar 50 μm, ×400). The intensity of METTL3 expression was higher in the tubular cell nuclear area of kidney tissue from the CKD patients than in that from the control patients (Fig. 8b). The proportion of the METTL3-positive area was compared according to disease, CKD stage, proteinuria level, and degree of interstitial fibrosis. METTL3 expression in CKD patients, especially those with IgA nephropathy, was significantly higher than that in the control patients (*P* = 0.013; Fig. 8b). In addition, patients with heavy proteinuria and higher degrees of interstitial fibrosis had larger areas of METTL3 positivity (Fig. 8b). These results suggest a role for METTL3-mediated m^6^A modifications in human CKD.

## Discussion

We investigated alterations in RNA methylation and the inhibitory effects of METTL3 on the development of kidney fibrosis in UUO-induced model mice and TGF-β-challenged human kidney tubular epithelial cells. In addition, we examined METTL3 expression in the kidney tissues of CKD patients. We demonstrated that the mRNA and protein levels of m^6^A methyltransferases (writers), demethylases (erasers), and m^6^A-binding proteins (readers) are related to RNA methylation changes during kidney fibrosis. Our experimental results showed that inhibition of METTL3 can suppress EMT and reduce the development and progression of kidney fibrosis both in vitro and in vivo. Furthermore, using MeRIP-seq, we found that *NET1* could be a possible regulator of TGF-β-mediated kidney cell fibrosis in UUO mouse models. Finally, we showed that the METTL3 expression levels in kidney tissues collected from patients with CKD were higher than those in controls. These results suggest that changes in RNA methylation, especially activation of METTL3, play an important role in kidney fibrosis and that the initiation and progression of kidney fibrosis can be alleviated through regulation of RNA methylation.

RNA methylation is one of the processes that controls gene expression at the post-transcriptional level^6^. RNA methylation is widespread in mRNA, transfer RNA (tRNA), ribosomal RNA (rRNA), noncoding small RNA (sncRNAs), and long-chain noncoding RNA (lncRNAs)^29^. m^6^A is the most common methylation of mRNA^30^. Proteins associated with RNA methylation include methylation-specific methyltransferases (writers), demethylases (erasers), and RNA methylation-recognition proteins (readers). The majority of m^6^A methylation on mRNA is carried out by protein complexes with METTL3 and METTL14^31^. METTL3 acts catalytically, whereas METTL14 aids in RNA substrate binding. As a major biological function of RNA methylation, RNA methylation can determine the survival of stem cells and regulate gene translation and the response to DNA damage^32^. Recent research has suggested that RNA methylation primarily regulates EMT^1^. EMT associated with RNA methylation contributes to the metastasis of cancer cells by decreasing E-cadherin expression^33^. In addition, RNA methylation is involved in solid organ fibrosis, including that in the lungs and liver^8–10,34,35^.

Recently, RNA methylation was suggested to be involved in kidney fibrosis. TGF-β increases m^6^A modification in HK-2 cells, and METTL3 suppression by knockdown attenuates TGF-β-induced EMT and fibrosis markers^36^. Ning et al. reported that the m^6^A RNA demethylase ALKBH5 was suppressed with an increase in the total m^6^A levels in a UUO-induced kidney fibrosis model and that soy isoflavone genistein administration restored ALKBH5 inhibition and improved kidney fibrosis by suppressing EMT and improving inflammatory markers^37^. ALKBH5 knockdown increased EMT and kidney fibrosis associated with α-smooth muscle actin and Snail expression. ALKBH5 overexpression increased E-cadherin expression and suppressed Snail expression. Liu et al. found that METTL3 methyltransferase, which mediates RNA m^6^A modification, is activated in the UUO model and that METTL3-induced HK-2 cell fibrosis is mediated by the miR-21-5p-activated SPRY1/ERK/NF-κB pathway^38^. Recently, Xu et al. reported that the METTL14-regulated PI3K/Akt signaling pathway contributes to the EMT of kidney tubular cells in diabetic kidney disease^39^. Our data highlight the role of METTL3 in kidney fibrosis models and suggest METTL3 as a therapeutic target for kidney fibrosis.

RNA m^6^A modification and activation of METTL3 have been suggested to be major mediators of kidney diseases in models other than TGF-β-induced and UUO kidney fibrosis. Meng et al. investigated the role of METTL3 in an ischemia‒reperfusion injury rat model and hypoxia/reoxygenation in vitro model. m^6^A and METTL3 expression levels increased in in vivo and in vitro experimental models, and METTL3 inhibition reduced m^6^A levels and cellular apoptosis^40^. In addition, cisplatin-induced acute kidney injury in mice led to increased m^6^A levels and alterations in the expression of methyltransferase complexes, including METTL3, METTL14, FTO, and ALKBH5^41^. In a mouse cystic disease model, METTL3 deletion suppressed cyst formation, and the methionine-METLL3-c-Myc/Camp pathway was involved in the pathogenesis of cystic kidney disease^42^. These data suggest that RNA methylation through METTL3 may have a protective effect against kidney disease through various mechanisms and pathways that are not directly related to kidney fibrosis.

NET1 is a specific guanine nucleotide exchange factor for RhoA and has been suggested to play a role in TGF-β signaling and EMT. Our data demonstrated that TGF-β treatment increased the mRNA expression of *NET1* and the m^6^A methylation of *NET1* mRNA (Fig. 5a, b). As TGF-β treatment increases and METTL3-specific inhibitors decrease the stability of *NET1* mRNA, m^6^A modification of *NET1* mRNA plays a role in the TGF-β-mediated increase in *NET1* expression by stabilizing mRNAs. In addition, *NET1* knockdown decreased the expression of fibrotic markers (Fig. 5e, f). m^6^A modification of *NET1* mRNA was also validated in UUO mouse models (Fig. 6e, f). Therefore, our data suggest that m^6^A modification of *NET1* mRNA is a contributing factor to kidney fibrosis.

In conclusion, RNA methylation mediated by METTL3 contributes to kidney fibrosis, and inhibition of RNA methylation attenuates kidney fibrosis in vitro in human kidney tubular epithelial cells and in vivo in mouse unilateral obstruction models. NET1 may be a regulator of TGF-β-mediated kidney cell fibrosis. Human kidney tissues from patients with CKD express higher levels of METTL3; therefore, modification of the METTL3-mediated RNA methylation pathway could be a therapeutic target for the development and progression of kidney fibrosis.

### Supplementary information

Supplementary Figures and Tables

## Data Availability

The main article and supplemental materials contain all the data related to this investigation.

## References

[1] GBD. (2020). Global, regional, and national burden of chronic kidney disease, 1990-2017: a systematic analysis for the Global Burden of Disease Study 2017. Lancet.

[2] Hill NR (2016). Global prevalence of chronic kidney disease—a systematic review and meta-analysis. PLoS ONE.

[3] Chen TK, Knicely DH, Grams ME (2019). Chronic kidney disease diagnosis and management: a review. J. Am. Med. Assoc..

[4] Wang C (2023). An update on renal fibrosis: from mechanisms to therapeutic strategies with a focus on extracellular vesicles. Kidney Res. Clin. Pract..

[5] Chuang PY, Menon MC, He JC (2013). Molecular targets for treatment of kidney fibrosis. J. Mol. Med..

[6] Zhou Y (2020). Principles of RNA methylation and their implications for biology and medicine. Biomed. Pharmacother..

[7] Luo J, Xu T, Sun K (2021). N6-methyladenosine RNA modification in inflammation: roles, mechanisms, and applications. Front. Cell Dev. Biol..

[8] Huang X, Lv D, Yang X, Li M, Zhang H (2020). m6A RNA methylation regulators could contribute to the occurrence of chronic obstructive pulmonary disease. J. Cell Mol. Med..

[9] Fan C (2021). Comprehensive analysis of the transcriptome-wide m6A methylation modification difference in liver fibrosis mice by high-throughput m6A sequencing. Front. Cell Dev. Biol..

[10] Yang L (2022). New advances of DNA/RNA methylation modification in liver fibrosis. Cell Signal.

[11] An JN (2019). Periostin induces kidney fibrosis after acute kidney injury via the p38 MAPK pathway. Am. J. Physiol. Renal Physiol..

[12] Varghese F, Bukhari AB, Malhotra R, De A (2014). IHC Profiler: an open source plugin for the quantitative evaluation and automated scoring of immunohistochemistry images of human tissue samples. PLoS ONE.

[13] Landini G, Martinelli G, Piccinini F (2021). Colour deconvolution: stain unmixing in histological imaging. Bioinformatics.

[14] Lv D (2022). PDGF signaling inhibits mitophagy in glioblastoma stem cells through N(6)-methyladenosine. Dev. Cell.

[15] Lin S, Choe J, Du P, Triboulet R, Gregory RI (2016). The m(6)A methyltransferase METTL3 promotes translation in human cancer cells. Mol. Cell.

[16] Andrews, S. *FastQC: A Quality Control Tool for High Throughput Sequence Data* (Babraham Bioinformatics, Babraham Institute, Cambridge, 2010).

[17] Bolger AM, Lohse M, Usadel B (2014). Trimmomatic: a flexible trimmer for Illumina sequence data. Bioinformatics.

[18] Dobin A (2013). STAR: ultrafast universal RNA-seq aligner. Bioinformatics.

[19] Li B, Dewey CN (2011). RSEM: accurate transcript quantification from RNA-Seq data with or without a reference genome. BMC Bioinforma..

[20] Meng J (2014). A protocol for RNA methylation differential analysis with MeRIP-Seq data and exomePeak R/Bioconductor package. Methods.

[21] Cui X (2016). Guitar: an R/Bioconductor package for gene annotation guided transcriptomic analysis of RNA-related genomic features. Biomed. Res. Int..

[22] Heinz S (2010). Simple combinations of lineage-determining transcription factors prime cis-regulatory elements required for macrophage and B cell identities. Mol. Cell.

[23] Blighe K., Rana S. & Lewis M. EnhancedVolcano: publication-ready volcano plots with enhanced colouring and labeling. https://bioconductor.org/packages/release/bioc/html/EnhancedVolcano.html (2018).

[24] Yankova E (2021). Small-molecule inhibition of METTL3 as a strategy against myeloid leukaemia. Nature.

[25] Bindea G (2009). ClueGO: a Cytoscape plug-in to decipher functionally grouped gene ontology and pathway annotation networks. Bioinformatics.

[26] Subramanian A (2005). Gene set enrichment analysis: a knowledge-based approach for interpreting genome-wide expression profiles. Proc. Natl. Acad. Sci. USA.

[27] Shen X (2001). The activity of guanine exchange factor NET1 is essential for transforming growth factor-beta-mediated stress fiber formation. J. Biol. Chem..

[28] Lee J, Moon HJ, Lee JM, Joo CK (2010). Smad3 regulates Rho signaling via NET1 in the transforming growth factor-beta-induced epithelial-mesenchymal transition of human retinal pigment epithelial cells. J. Biol. Chem..

[29] Statello L, Guo CJ, Chen LL, Huarte M (2021). Gene regulation by long non-coding RNAs and its biological functions. Nat. Rev. Mol. Cell Biol..

[30] Jiang X (2021). The role of m6A modification in the biological functions and diseases. Signal Transduct. Target Ther.

[31] He PC, He C (2021). m(6) A RNA methylation: from mechanisms to therapeutic potential. EMBO J..

[32] Xiang Y (2017). RNA m(6)A methylation regulates the ultraviolet-induced DNA damage response. Nature.

[33] Lin X (2019). RNA m(6)A methylation regulates the epithelial mesenchymal transition of cancer cells and translation of Snail. Nat. Commun..

[34] Ligresti G, Pham TX, Sanders YY (2022). Circular RNA methylation: a new twist in lung fibrosis. Am. J. Respir. Cell Mol. Biol..

[35] Wang S (2022). The combined effects of circular RNA methylation promote pulmonary fibrosis. Am. J. Respir. Cell Mol. Biol..

[36] Liu P (2020). m(6)A-induced lncRNA MALAT1 aggravates renal fibrogenesis in obstructive nephropathy through the miR-145/FAK pathway. Aging.

[37] Ning Y (2020). Genistein ameliorates renal fibrosis through regulation snail via m6A RNA demethylase ALKBH5. Front. Pharmacol..

[38] Liu E (2021). METTL3/N6-methyladenosine/ miR-21-5p promotes obstructive renal fibrosis by regulating inflammation through SPRY1/ERK/NF-kappaB pathway activation. J. Cell Mol. Med..

[39] Xu Z (2021). METTL14-regulated PI3K/Akt signaling pathway via PTEN affects HDAC5-mediated epithelial-mesenchymal transition of renal tubular cells in diabetic kidney disease. Cell Death Dis..

[40] Meng F (2020). METTL3 contributes to renal ischemia-reperfusion injury by regulating Foxd1 methylation. Am. J. Physiol. Renal Physiol..

[41] Li CM, Li M, Zhao WB, Ye ZC, Peng H (2021). Alteration of N6-methyladenosine RNA profiles in cisplatin-induced acute kidney injury in mice. Front. Mol. Biosci..

[42] Ramalingam H (2021). A methionine-Mettl3-N(6)-methyladenosine axis promotes polycystic kidney disease. Cell. Metab..

