# Supplementary material for: Targeting the m6A RNA methyltransferase METTL3 attenuates the development of kidney fibrosis
Source: Exp Mol Med. 2024 Feb 1;56(2):355–69. doi: 10.1038/s12276-024-01159-5 (PMC10907702; doi:10.1038/s12276-024-01159-5)
Supplement: Supplementary file 1 — Supplementary Figures and Tables [file 12276_2024_1159_MOESM1_ESM.pdf]

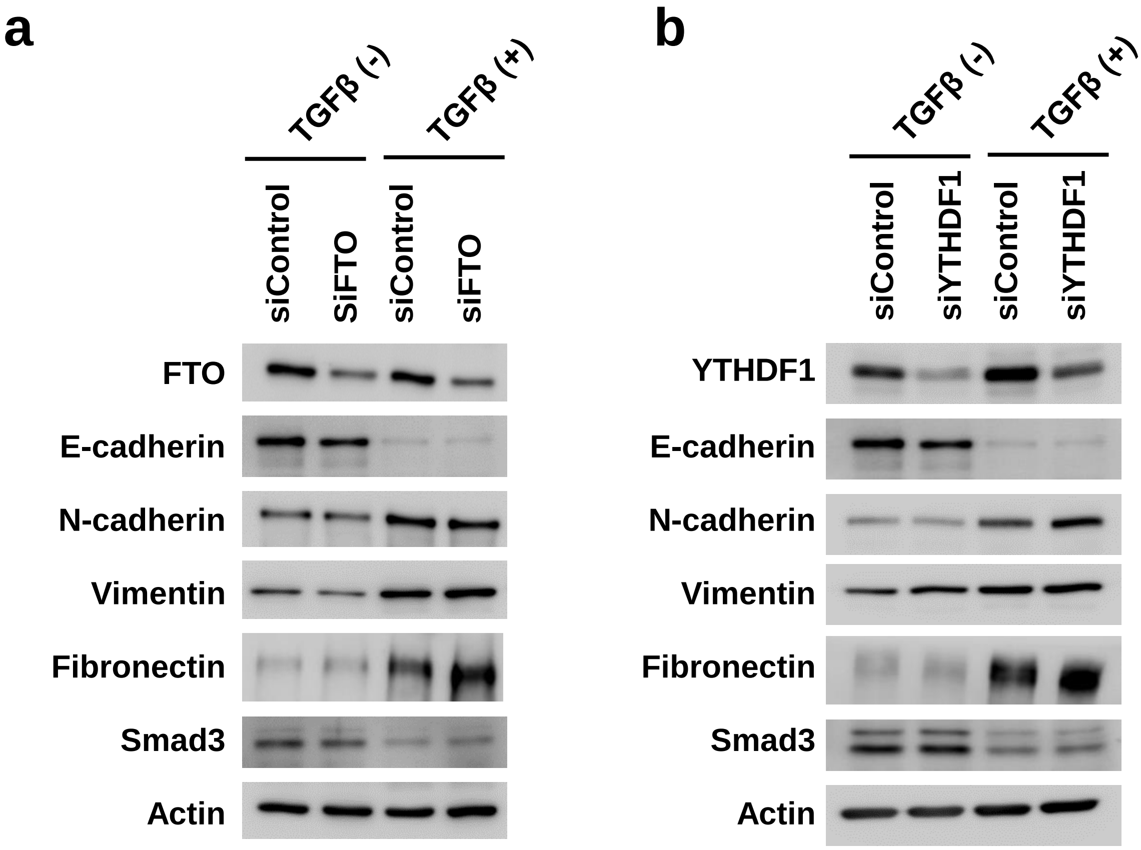

Supplementary Fig. 1. Knock-down effect of FTO and YTHDF1 on TGFβ-induced expression of epithelial-mesenchymal transition markers in renal tubular epithelial cells.

(a, b) Western blots for inhibitory effects of RNA methylation related protein including FTO (a) and YTHDF1 (b). After RNA methylation related proteins were inhibited using siFTO (a) and siYTHDF1 (b), HK-2 cells were challenged with TGFβ (10 ng/ml for 48 hours).

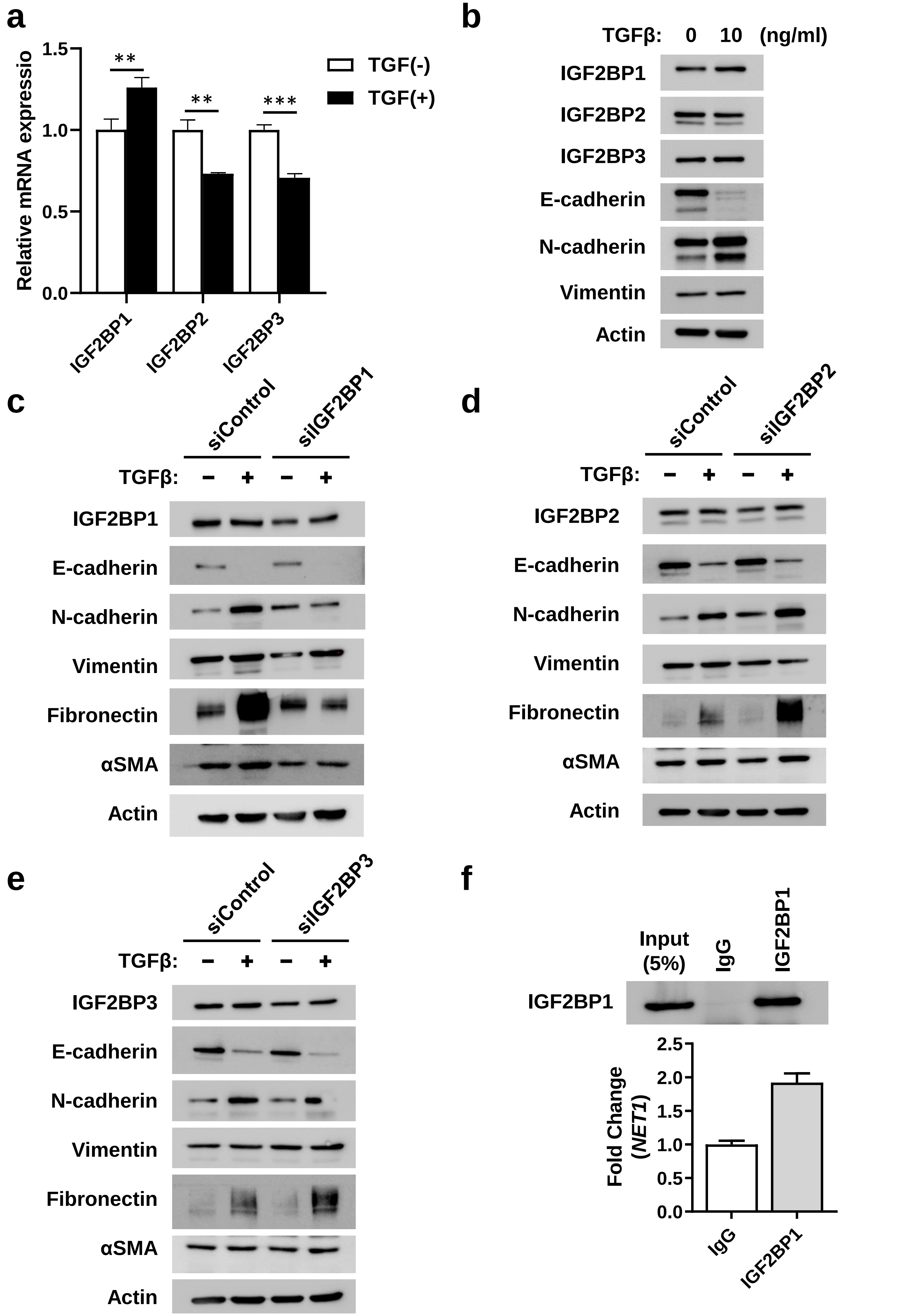

a
Relative mRNA expressio
1.5
1.0
0.5
0.0
**
**
***
TGF(-)
TGF(+)
IGF2BP1
IGF2BP2
IGF2BP3
b
TGFβ: 0 10 (ng/ml)
IGF2BP1
IGF2BP2
IGF2BP3
E-cadherin
N-cadherin
Vimentin
Actin
c
siControl
siIGF2BP1
TGFβ: − + − +
IGF2BP1
E-cadherin
N-cadherin
Vimentin
Fibronectin
αSMA
Actin
d
siControl
siIGF2BP2
TGFβ: − + − +
IGF2BP2
E-cadherin
N-cadherin
Vimentin
Fibronectin
αSMA
Actin
e
siControl
siIGF2BP3
TGFβ: − + − +
IGF2BP3
E-cadherin
N-cadherin
Vimentin
Fibronectin
αSMA
Actin
f
Input (5%)
IgG
IGF2BP1
IGF2BP1
Fold Change (NET1)
2.5
2.0
1.5
1.0
0.5
0.0
IgG
IGF2BP1

Supplementary Fig. 2. Effect of IGF2BPs on TGFβ-induced expression of epithelial-mesenchymal transition markers in renal tubular epithelial cells.

(a) Messenger RNA levels of IGF2BPs with or without TGF-β challenge (10 ng/mL for 48 h) analyzed using real time-PCR. Comparison of means among two groups was analyzed using t-test. $^{**}P < 0.01$, $^{***}P < 0.001$. **(b)** Western blot of HK-2 cells for IGF2BPs with TGF-β challenge (10 ng/mL) of 48 h. **(c-e)** Knockdown effect of IGF2BP1 (c), IGF2BP2 (d), and IFG2BP3 (e) on TGF-β-mediated gene expression of fibrosis-related genes. After IGF2BPs were inhibited using siRNAs, HK-2 cells were challenged with TGF-β (10 ng/mL for 48 h). **(f)** The binding of *NET1* mRNA to IGF2BP1 estimated by RNA immunoprecipitation (RIP)-qPCR for IGF2BP1.

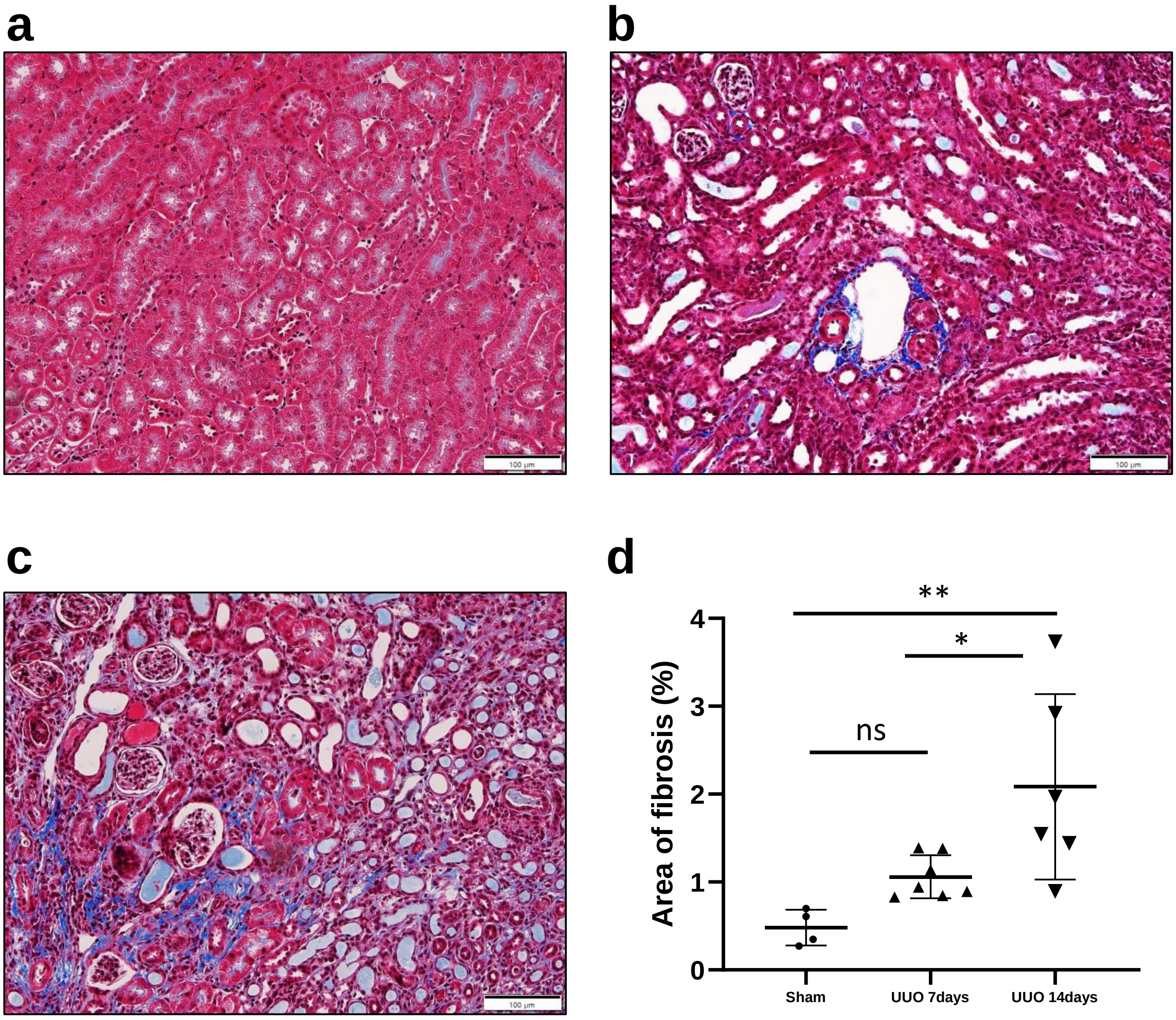

Supplementary Fig. 3. Generation of unilateral ureteral obstruction (UUO) mouse model and Masson's trichrome staining for fibrosis.

(a-c) Representative images of Masson's trichrome staining for kidney tissues from Sham (a), UUO 7 days (b), and UUO 14 days (c). **(d)** Measurement of Masson's trichrome staining for fibrosis. Comparison of means among three groups was analyzed using ANOVA with post-hoc Tukey test respectively. $^{ns}P > 0.05$, $^{*}P < 0.05$, $^{**}P < 0.01$, $^{***}P < 0.001$.

a

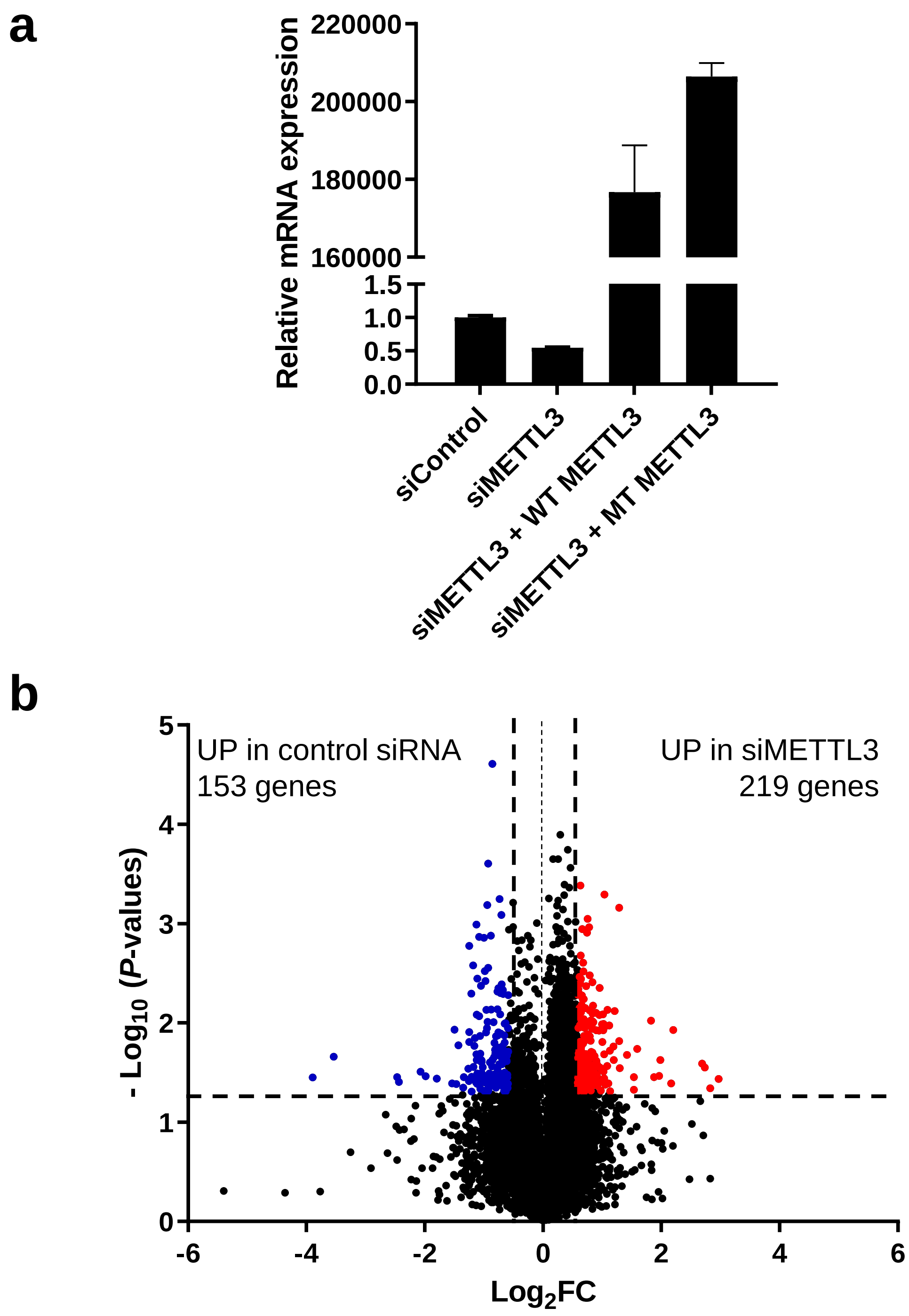

Supplementary Fig. 4. Transcriptomic profiles of METTL3 knock-down effect in renal tubular epithelial cells.

(a) Knock-down or overexpression of *METTL3* in HK2 cells. After transfection of siRNA targeting 3'UTR of *METTL3* (siMETTL3) or METTL3-overexpressing vectors, mRNA levels of *METTL3* were analyzed using real time-PCR. WT: wild-type, MT: mutant. **(b)** Volcano plot for differentially expressed gene from RNA sequencing between control and METTL3 siRNA-treated HK2 cells ($P < 0.05$, |fold change| ≥ 1.5) in the presence of TGFβ (10 ng/ml for 48 hours). X-axis represents fold change of METTL3 siRNA (siMETTL3)-treated cells compared with control siRNA-treated cells.

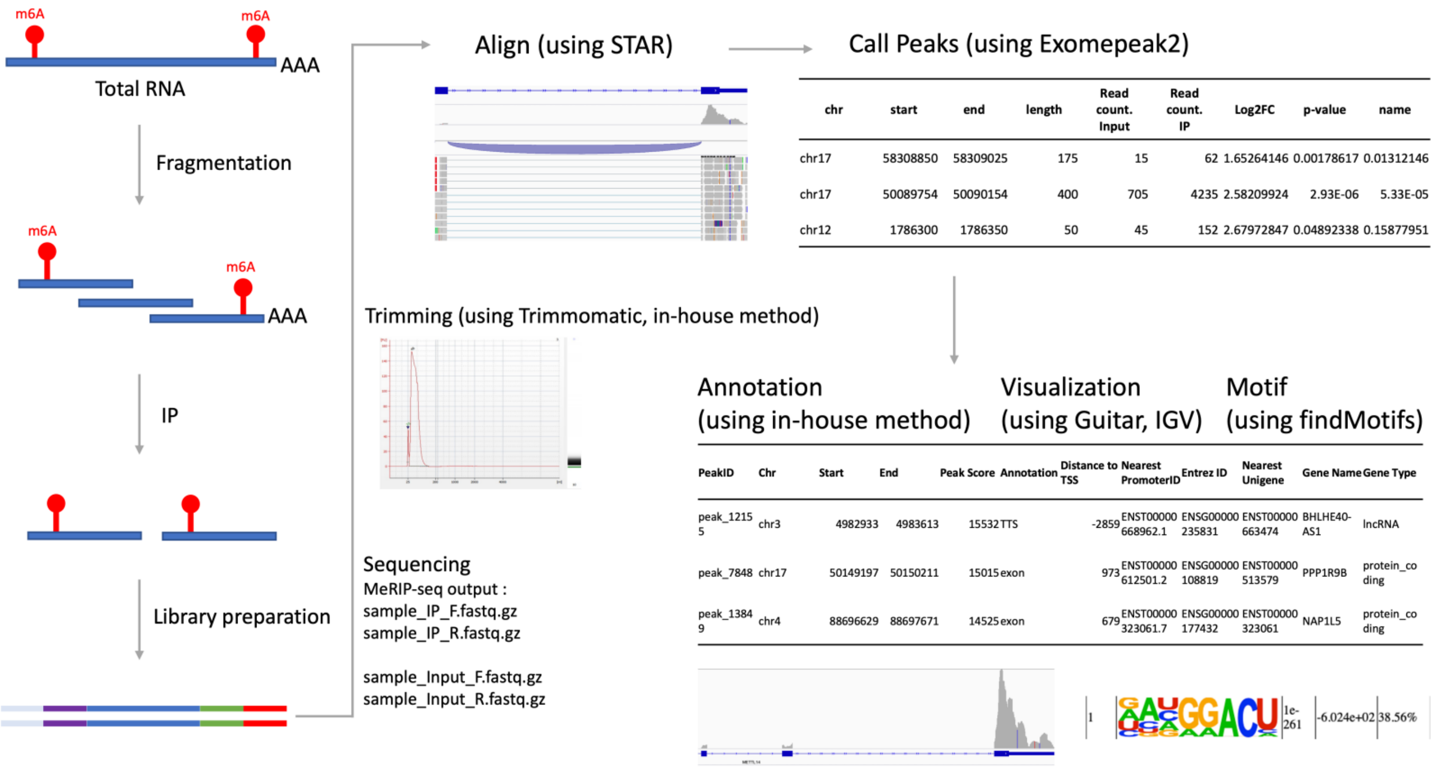

Supplementary Fig. 5. Bioinformatic pipeline for the analysis of MeRIP-seq

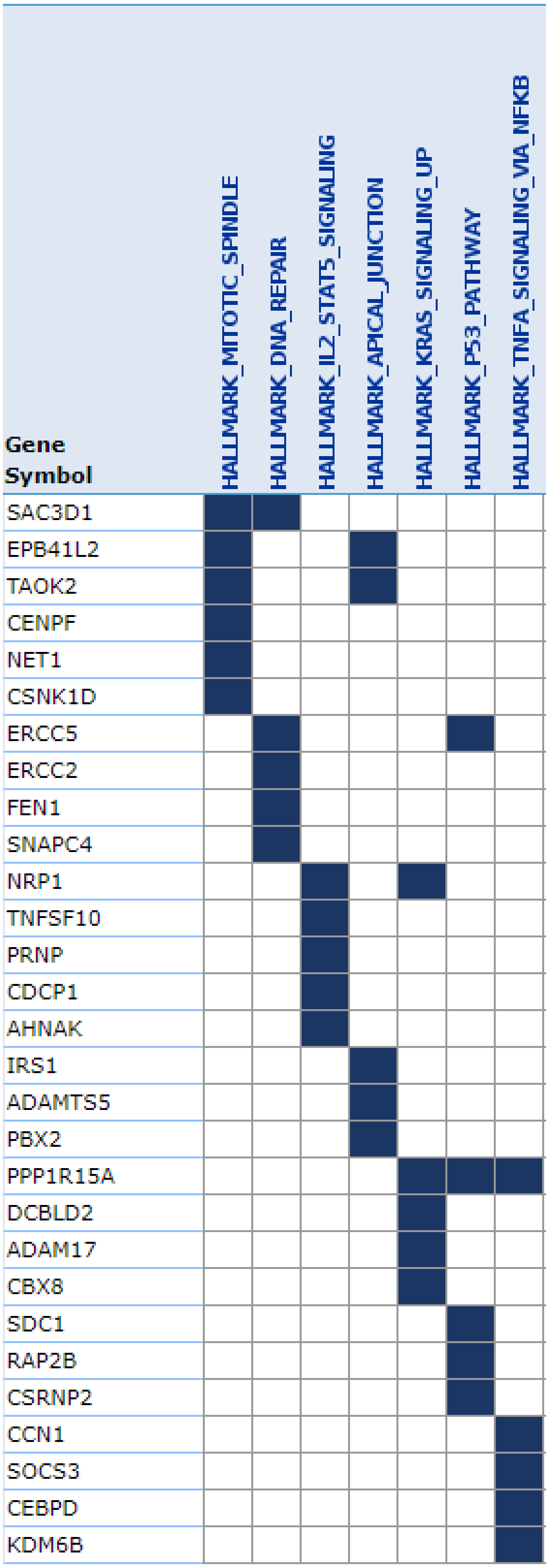

Supplementary Fig. 6. Gene set analysis of differentially methylated genes by TGFβ treatment in HK cells.

Heat map represents the significantly enriched hallmark gene sets ($P < 0.01$) in 261 genes with differentially methylated m6A peaks between control and TGFβ-treated HK2 cells. Each row represents a gene and each column represents a gene set. Blue indicates the genes that are included in each gene set.

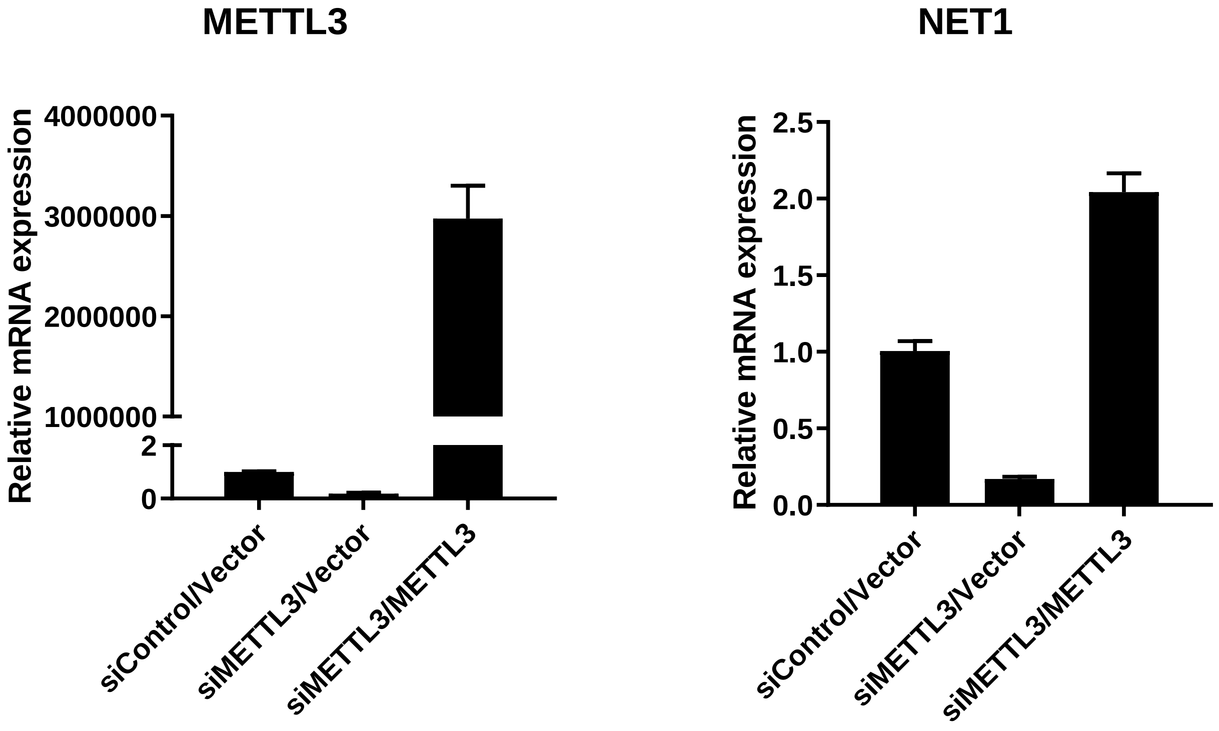

Supplementary Fig. 7. Effect of *METTL3* overexpression on the mRNA levels of *NET1*.

After transfection of siRNA targeting 3’UTR of *METTL3* (siMETTL3) and pcDNA-3xFlag-METTL3 vector (resistant for NET1 siRNA) for 24h, mRNA levels of *METTL3* and *NET1* were analyzed using real time-PCR.

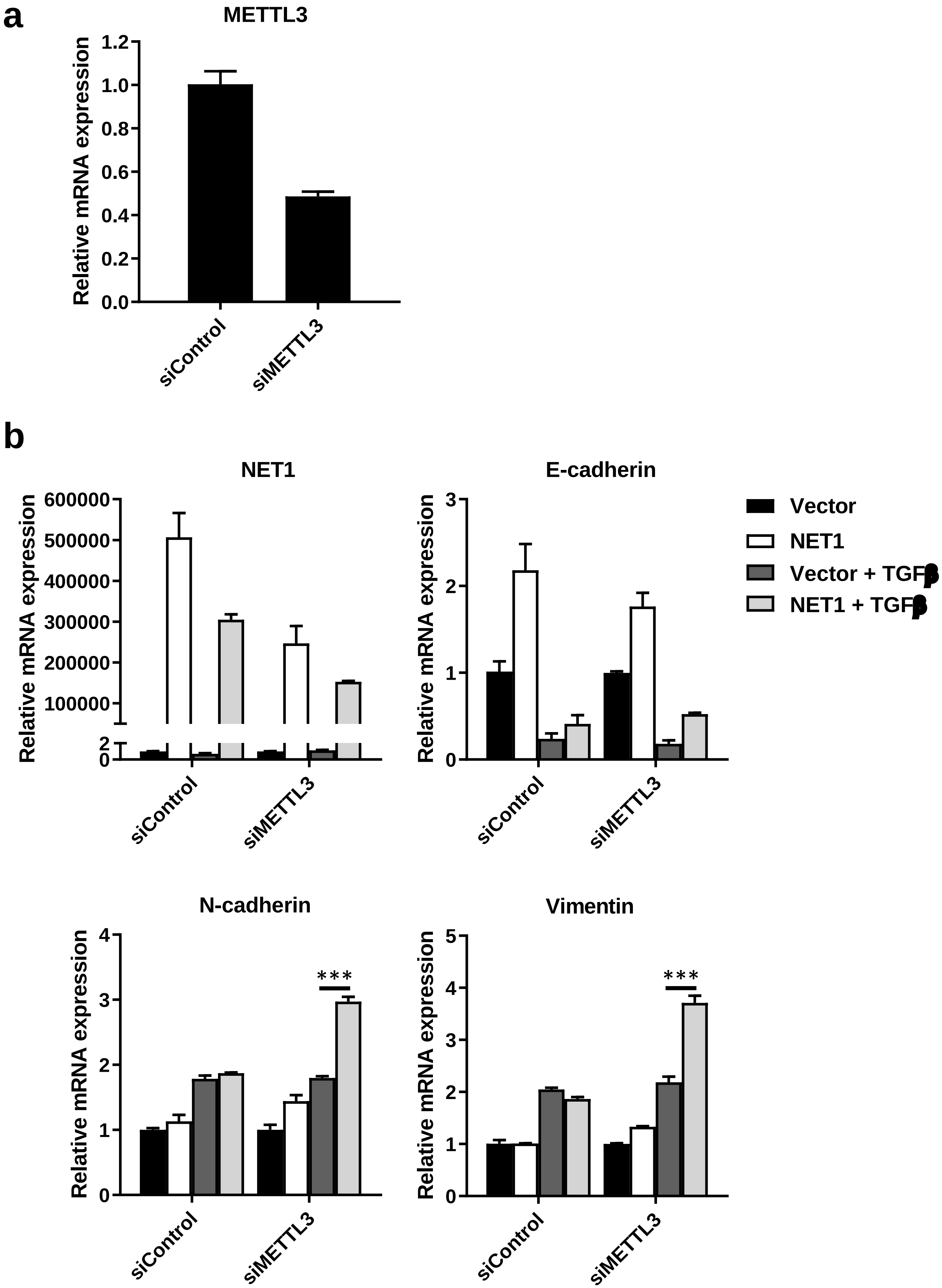
a
METTL3
Relative mRNA expression
1.2
1.0
0.8
0.6
0.4
0.2
0.0
siControl
siMETTL3
b
NET1
600000
500000
400000
300000
200000
100000
2
0
E-cadherin
3
2
1
0
Vector
NET1
Vector + TGFβ
NET1 + TGFβ
N-cadherin
4
3
2
1
0
***
Vimentin
5
4
3
2
1
0
***

Supplementary Fig. 8. Effect of *NET1* overexpression on TGF-β-induced epithelial-to-mesenchymal transition (EMT).

(a) Knock-down of *METTL3* in HK2 cells. After transfection of siRNA targeting 3’UTR of *METTL3* (siMETTL3), mRNA levels of *METTL3* were analyzed using real time-PCR. **(b)** The effect of *NET1* overexpression on TGF-β-induced EMT. After transfection of *NET1*-overexpressing vector with control siRNA or METTL3 siRNA, the expression of EMT markers after TGF-β treatment (10 ng/mL for 48 h) was evaluated using real-time PCR. $^{***}P < 0.001$.

a

NAME	SIZE	ES	NES	NOM p-val	FDR q-val
KEGG_HYPERTROPHIC_CARDIOMYOPATHY_HCM	70	-0.95265	-1.72015	0	0.012103
KEGG_DILATED_CARDIOMYOPATHY	77	-0.9539	-1.6955	0	0.023984
KEGG_PATHOGENIC_ESCHERICHIA_COLI_INFECTION	52	-0.93595	-1.65518	0.003328	0.081588
KEGG_SMALL_CELL_LUNG_CANCER	81	-0.8899	-1.6022	0.009124	0.143131
KEGG_LEUKOCYTE_TRANSENDOTHELIAL_MIGRATION	105	-0.87422	-1.641	0.009381	0.094433
KEGG_ARRHYTHMOGENIC_RIGHT_VENTRICULAR_CARDIOMYOPATHY_ARVC	64	-0.92263	-1.62461	0.011364	0.110632
KEGG_OLFACTORY_TRANSDUCTION	53	-0.89076	-1.57099	0.014652	0.156411
KEGG_VIBRIO_CHOLERAE_INFECTION	51	-0.90992	-1.59467	0.014787	0.140154
KEGG_PATHWAYS_IN_CANCER	302	-0.75512	-1.5536	0.015444	0.178893
KEGG_ADHERENS_JUNCTION	73	-0.88774	-1.58555	0.015986	0.144525
KEGG_GAP_JUNCTION	81	-0.84924	-1.54688	0.019892	0.178955
KEGG_FOCAL_ADHESION	186	-0.76115	-1.49337	0.024621	0.25852
KEGG_TASTE_TRANSDUCTION	32	-0.91756	-1.53268	0.025547	0.182106
KEGG_TIGHT_JUNCTION	120	-0.82151	-1.53786	0.03964	0.183961
KEGG_VASCULAR_SMOOTH_MUSCLE_CONTRACTION	92	-0.81379	-1.49197	0.047706	0.245274

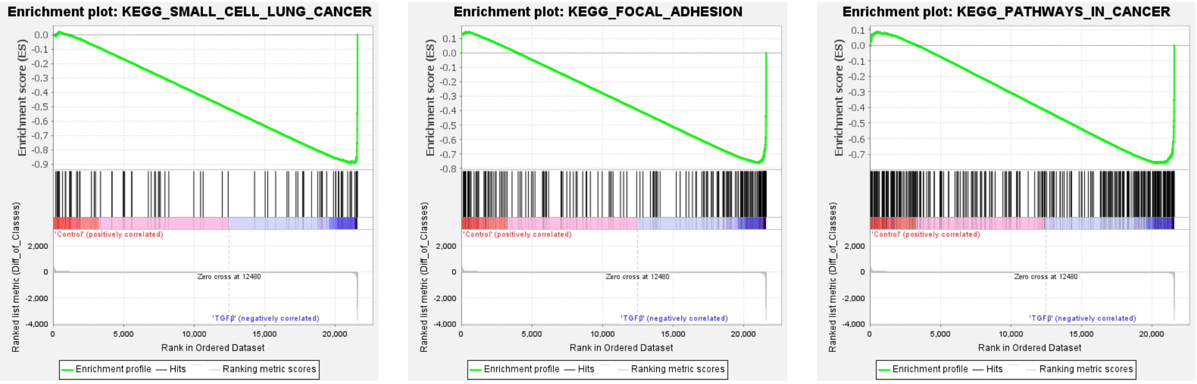

b

NAME	SIZE	ES	NES	NOM p-val	FDR q-val
KEGG_SMALL_CELL_LUNG_CANCER	81	0.803663	1.526731	0.044776	0.455755
KEGG_FOCAL_ADHESION	190	0.719876	1.425551	0.065954	0.59296
KEGG_PATHWAYS_IN_CANCER	302	0.658413	1.352944	0.089501	0.615103
KEGG_VASCULAR_SMOOTH_MUSCLE_CONTRACTION	89	0.726953	1.35644	0.134438	0.635671
KEGG_DILATED_CARDIOMYOPATHY	78	0.684036	1.268354	0.184701	0.707141
KEGG_HYPERTROPHIC_CARDIOMYOPATHY_HCM	72	0.664694	1.214385	0.219466	0.742835
KEGG_VIBRIO_CHOLERAE_INFECTION	49	0.643359	1.186104	0.264479	0.762835
KEGG_LEUKOCYTE_TRANSENDOTHELIAL_MIGRATION	104	0.584093	1.108134	0.325279	0.858101
KEGG_OLFACTORY_TRANSDUCTION	47	0.581169	1.051948	0.395669	0.879523
KEGG_ADHERENS_JUNCTION	73	0.554115	1.047062	0.401079	0.869462
KEGG_ARRHYTHMOGENIC_RIGHT_VENTRICULAR_CARDIOMYOPATHY_ARVC	66	0.536396	1.002239	0.449198	0.888991

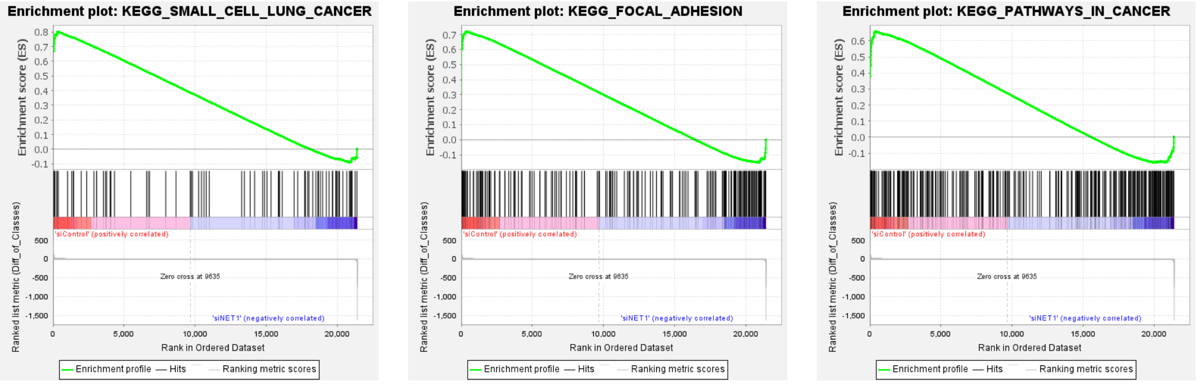

Supplementary Fig. 9. Gene Set Enrichment Analysis (GSEA) using KEGG pathways in HK2 control cells, TGFβ-treated cells, and TGFβ-treated cells with NET1 knock-down.

(a) GSEA between HK2 control and TGFβ-treated cells using KEGG pathway. Upper table shows the list of significantly enriched gene sets in TGFβ-treated cells compared to control cells ($P < 0.05$). ES: enrichment score, NES: normalized enrichment score, NOM p-val: nominal P-value, FDR q-val: false discovery rate Q-value. Lower plots show the enrichment plots of representative gene sets enriched in TGFβ-treated cells. On the x-axis, the genes are ranked from the most upregulated to the most downregulated between control (left end; positively correlated) and TGFβ-treated cells (right end; negatively correlated) treatment. The y-axis shows a running enrichment score for control cells. **(b)** GSEA between HK2 TGFβ-treated cells and TGFβ-treated NET1 knock-down cells using KEGG pathway. Upper table shows the list of enriched gene sets in TGFβ-treated cells compared to TGFβ-treated NET1 knock-down cells, out of 15 gene sets from A (NES > 1). ES: enrichment score, NES: normalized enrichment score, NOM p-val: nominal P-value, FDR q-val: false discovery rate Q-value. Lower plots show the enrichment plots of representative gene sets enriched in TGFβ-treated cells. On the x-axis, the genes are ranked from the most upregulated to the most downregulated between TGFβ-treated cells (left end; positively correlated) and TGFβ-treated NET1 knock-down cells (right end; negatively correlated) treatment. The y-axis shows a running enrichment score for TGFβ-treated cells.

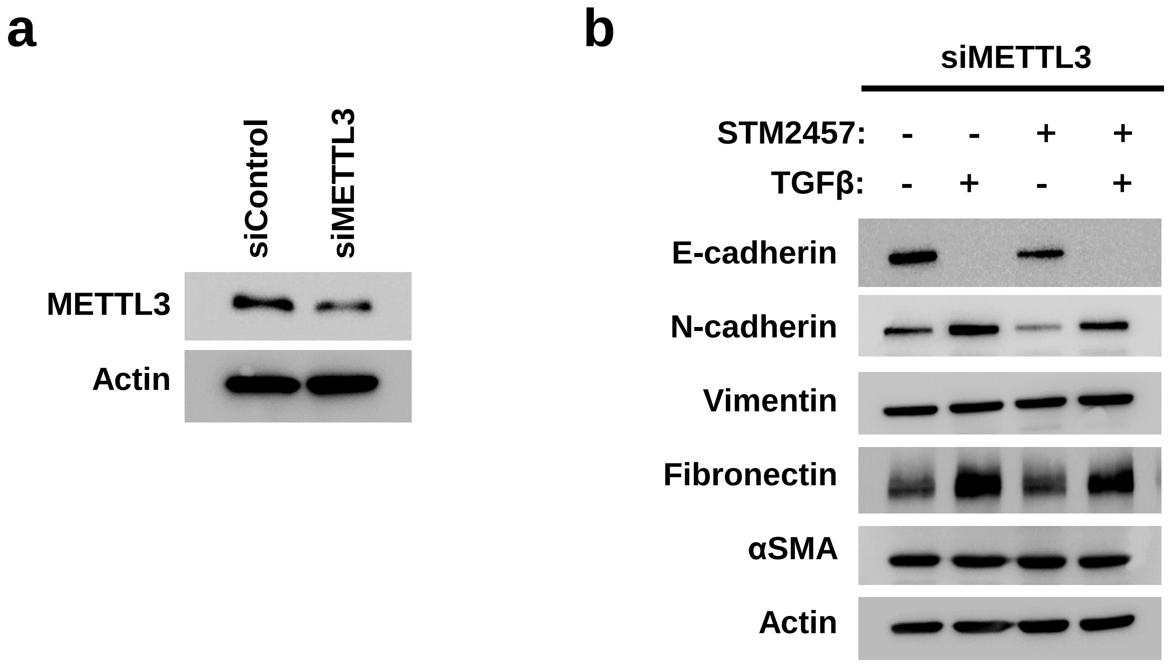

Supplementary Fig. 10. Effect of METTL3 knock-down on the effect of STM2457 in TGF-β-induced epithelial-to-mesenchymal transition (EMT).

(a) Knock-down of METTL3 in HK2 cells. After transfection of siRNA targeting 3'UTR of *METTL3* (siMETTL3), protein levels of METTL3 were analyzed using western blot. **(b)** Western blot of METTL3 knocked-down HK-2 cells challenged with TGF-β (10 ng/mL for 48 h) for fibrosis-related proteins with or without STM2457 (5 μM for 48 h).

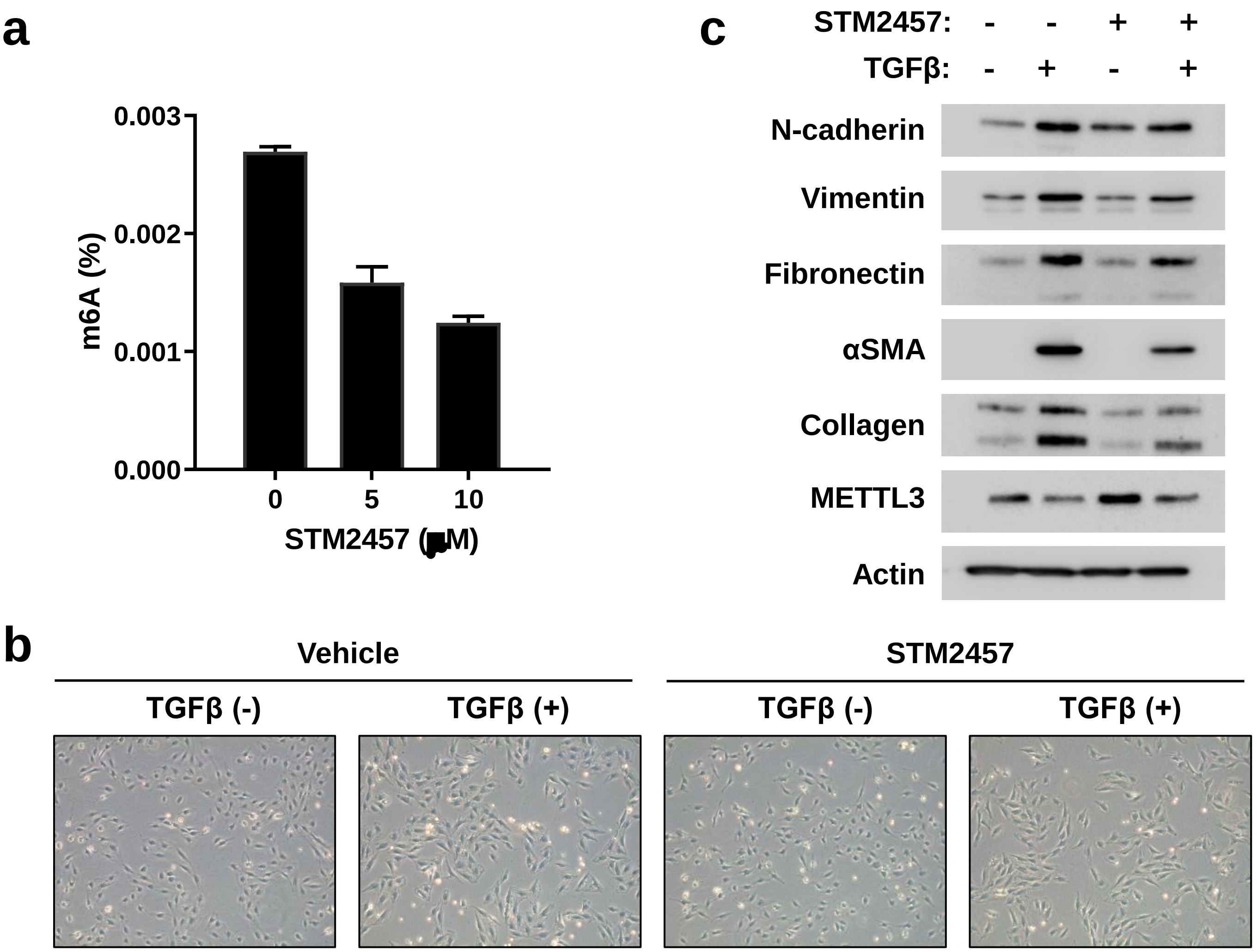

Supplementary Fig. 11. Effect of a METTL3 inhibitor on kidney fibroblasts.

(a) Total RNA N^6-methyladenosine (m6A) levels in rat kidney fibroblast, NRK49F cells after treatment with a METTL3 inhibitor, STM2457 (5, and 10 μM for 24 hours). **(b)** Representative microscopic images for NRK49F cells after treatment of TGFβ (10 ng/ml for 48 hours) with or without STM2457 (5 μM for 48 hours). **(c)** Western blot of NRK49F cells challenged with TGFβ (10 ng/ml for 48 hours) for fibrosis-related proteins with or without STM2457 (5 μM for 48 hours).

Supplementary Table 1. List of primary antibodies for western blot.

Protein	Molecular weight	Company	Cat. No.
METTL3	70	Cell Signaling Technology	#86132
METTL14	65	Cell Signaling Technology	#51104
METTL16	78	Cell Signaling Technology	#17676
ALKBH5	52	Millipore	#ABE547
FTO	60	Cell Signaling Technology	#45980
YTHDF1	70	Cell Signaling Technology	#86463
YTHDF2	62	Cell Signaling Technology	#80014
YTHDF3	64	Proteintech	25537-1-AP
E-Cadherin	135	Cell Signaling Technology	#3195
N-Cadherin	140	Cell Signaling Technology	#13116
αSMA	42	Abcam	ab5694
Vimentin	57	Cell Signaling Technology	#5741
Fibronectin	220	Santacruz	sc-8422
CollagenI	115 120	Abcam	ab21286
NET1	54	Santacruz	sc-271941

Supplementary Table 2. Primer list for real-time PCR

Gene	Species	Forward or reverse	Sequence	Reference
METTL3	Human	F	5′-CTA TCT CCT GGC ACT CGC AAG A-3′	
		R	5′-GCT TGA ACC GTG CAA CCA CAT C-3′	
YTHDF1	Human	F	5′- CAA GCA CAC AAC CTC CAT CTT CG-3′	
		R	5′-GTA AGA AAC TGG TTC GCC CTC AT-3′	
ALKBH5	Human	F	5’-TCCAGTTCAAGCCTATTCG-3’	
		R	5’-CATCTAATCTTGTCTTCCTGAG-3’	
FTO	Human	F	5'-TGGTGTCCCAAGAAATCGTG-3'	
		R	5'-TGCAGGCCGTGAACCAC-3'	
METTL14	Human	F	5’-AGAAACTTGCAGGGCTTCCT-3’	
		R	5’-TCTTCTTCATATGGCAAATTTTCTT-3	
METTL16	Human	F	5’-ACAGAAGACACTCCTGATGG-3’	
		R	5’-TTAACAGAACTAGGCGGAGG-3’	
YTHDF2	Human	F	5’-CCTTAGGTGGAGCCATGATTG-3’	
		R	5’-TCTGTGCTACCCAACTTCAGT-3’	
YTHDF3	Human	F	5’-TGACAACAAACCGGTTACCA-3’	
		R	5’-TGTTTCTATTTCTCTCCCTACGC-3’	
NET1	human	F	5'-CTG TTC ACC TCG GGA CAT TT-3'	Anticancer Res. 2014 Mar;34(3):1321-31
		R	5'-TGG AGC TGT CAG ACG TTT TG-3'	
Mettl3	Mouse	F	5'-CTGGGCACTTGGATTTAAGGAA-3’	EMBO Rep (2020)21:e49229https://doi.org/10.15252/embr.201949229
		R	5'-TGAGAGGTGGTGTAGCAACTT-3’	
Mettl14	Mouse	F	5'-CTGAGAGTGCGGATAGCATTG-3’	
		R	5'-GAGCAGATGTATCATAGGAAGCC-3’	
Ythdf1	Mouse	F	5'-ACAGTTACCCCTCGATGAGTG-3’	
		R	5'-GGTAGTGAGATACGGGATGGGA-3’	
Ythdf2	Mouse	F	5'-GAGCAGAGACCAAAAGGTCAAG-3’	
		R	5'-CTGTGGGCTCAAGTAAGGTTC-3’	
Ythdf3	Mouse	F	5'-CATAGGGCAACAGAGGAAACAG-3’	
		R	5'-ATCTCCAGCCGTGGACCAT-3’	
Fto	Mouse	F	5'-TTCATGCTGGATGACCTCAATG-3’	
		R	5'-GCCAACTGACAGCGTTCTAAG-3’	
Alkbh5	Mouse	F	5'-CGCGGTCATCAACGACTACC-3’	
		R	5'-ATGGGCTTGAACTGGAACTTG-3’	
α-Sma	Mouse	F	5'-GGCTCTGGGCTCTGTAAGG	December 2018, International Journal of Molecular Sciences 19(12):4114
		R	5'-CTCTTGCTCTGGGCTTCATC	
Col1a1	Mouse	F	5'-ACCTGTGTGTTCCCTACTCA	
		R	5'-GACTGTTGCCTTCGCCTCTG	
Fibronectin	Mouse	F	5'-ATGACGATGGGAAGACCTAC	
		R	5'-GGCTGGAAAGATTACTCTCG	
E-cadherin	Mouse	F	5′-AATGGCGGCAATGCAATCCCAAGA-3′	Phytomedicine
		R	5′-TGCCACAGACCGATTGTGGAGATA-3'	Volume 23, Issue 13, 1 December 2016, Pages 1680-1690
N-cadherin	Mouse	F	5′-TGGAGAACCCCATTGACATT-3′	
		R	5′-TGATCCCTCAGGAACTGTCC-3′	
Vimentin	Mouse	F	5'-TTCTCTGGCACGTCTTGACC-3'	Frontiers in Pharmacology, Feb-2018, volume 9, Article 72
		R	5'- CTCCTGGAGGTTCTTGGCAG-3'	
Net1	Mouse	F	5'- ACATTCTCGTGAACTGGTTA-3'	Molecular Endocrinology 28: 1948–1960, 2014
		R	5'-GCTGGAGGAAGTCTTGGA-3'	
rNet1	Rat	F	5’-ATTGTCTGGCTGAACCAGAGG-3’	
		R	5’-TGCAGGTATGAGAAACCAAAGC-3’	
rGapdh	Rat	F	5’-ATGGCCTTCCGTGTTCCTACCC-3’	
		R	5’-GCCTGCTTCACCACCTTCTTGATG-3’	
NET1-MeRIP	Human	F	5’-CGCTTACAGATGTGGCTCTG-3’	
		R	5’-ATGATGCTCCCCTTACGAGA-3’	
Net1_MeRIP	Mouse	F	5‘-CCCCATCAGGGAATACACAC-3’	
		R	5‘-TGGATTGTGGCTCCAGTGTA-3’	

Supplementary Table 3. List of siRNA for gene knock-down

Gene	Company	Cat. No. / Sequence	Reference
YTHDF1 siRNA	Santacruz	sc-76945	
ALKBH5 siRNA	Santacruz	sc-93856	
FTO siRNA	Santacruz	sc-75002	
METTL3 siRNA	Sense	GCUGCACUUCAGACGAAUUAUCAAT	Open Med (Wars). 2019 Mar 2;14:25-31
	Anti-sense	AUUGAUAAUUCGUCUGAAGUGCAGCUU	
METTL3 siRNA for 3' UTR	Sense	CUAAACCUGAAGAGUGAUAUUUGTA	
	Anti-sense	UACAAAUAUCACUCUUCAGGUUUAGCU	
